# The RAB11A-Positive Compartment Is a Primary Platform for Autophagosome Assembly Mediated by WIPI2 Recognition of PI3P-RAB11A

**DOI:** 10.1016/j.devcel.2018.03.008

**Published:** 2018-04-09

**Authors:** Claudia Puri, Mariella Vicinanza, Avraham Ashkenazi, Matthew J. Gratian, Qifeng Zhang, Carla F. Bento, Maurizio Renna, Fiona M. Menzies, David C. Rubinsztein

**Affiliations:** 1 Department of Medical Genetics, Wellcome Trust/MRC Building, Cambridge Biomedical Campus, Hills Road, Cambridge CB2 0XY, UK; 2 Cambridge Institute for Medical Research, Wellcome Trust/MRC Building, Cambridge Biomedical Campus, Hills Road, Cambridge CB2 0XY, UK; 3 UK Dementia Research Institute, Wellcome Trust/MRC Building, Cambridge Biomedical Campus, Hills Road, Cambridge CB2 0XY, UK; 4 Babraham Institute, Babraham Research Campus, Cambridge CB22 3AT, UK

**Keywords:** autophagy, recycling endosome, autophagy platform, RAB11A, mitophagy, transferrin receptor, macroautophagy, WIPI2

## Abstract

Autophagy is a critical pathway that degrades intracytoplasmic contents by engulfing them in double-membraned autophagosomes that are conjugated with LC3 family members. These membranes are specified by phosphatidylinositol 3-phosphate (PI3P), which recruits WIPI2, which, in turn, recruits ATG16L1 to specify the sites of LC3-conjugation. Conventionally, phosphatidylinositides act in concert with other proteins in targeting effectors to specific membranes. Here we describe that WIPI2 localizes to autophagic precursor membranes by binding RAB11A, a protein that specifies recycling endosomes, and that PI3P is formed on RAB11A-positive membranes upon starvation. Loss of RAB11A impairs the recruitment and assembly of the autophagic machinery. RAB11A-positive membranes are a primary direct platform for canonical autophagosome formation that enables autophagy of the transferrin receptor and damaged mitochondria. While this compartment may receive membrane inputs from other sources to enable autophagosome biogenesis, RAB11A-positive membranes appear to be a compartment from which autophagosomes evolve.

## Highlights

• RAB11A binds WIPI2 via a conserved RAB11-binding domain and regulates autophagy
• Proteins regulating autophagosome formation localize on RAB11A-positive compartment
• Transferrin receptor is an autophagy substrate recruited to forming autophagosomes
• Damaged mitochondria are engulfed by RAB11A-positive compartment

## Graphical Abstract

**Figure:**
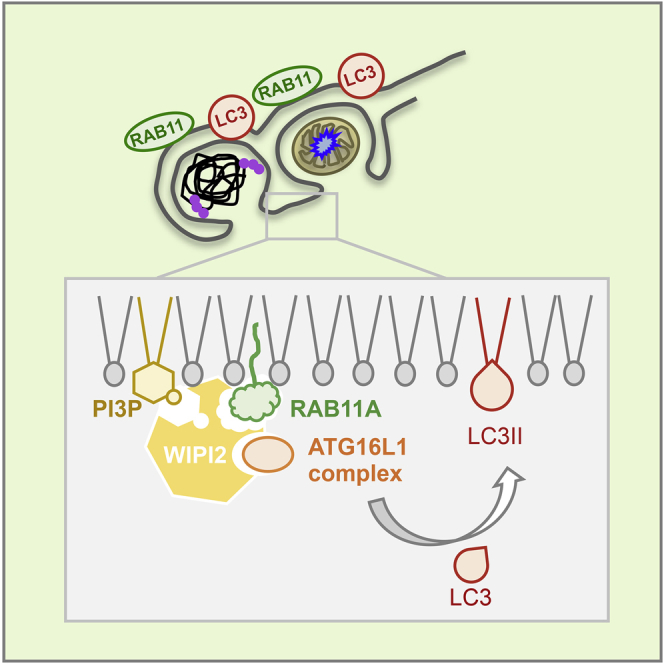

## Teaser

Puri et al. report that key events preceding completion of autophagosome formation occur on a RAB11A- and transferrin receptor-positive membrane compartment, likely the recycling endosome. RAB11A recruits WIPI2 for ATG16L-mediated LC3 conjugation, suggesting that this RAB11A compartment, beyond contributing membrane to autophagosomes, is a primary platform on which autophagosomes form.

## Introduction

Macroautophagy (henceforth autophagy) is a critical pathway that degrades intracytoplasmic contents by engulfing them in double-membrane vesicles, called autophagosomes, which are then trafficked to lysosomes for degradation. Multiple autophagosomes are continually formed at distinct sites in mammalian cells and have half-lives of around 30 min before being degraded (Fass et al., 2006). The first recognizable structure associated with autophagy is the cup-shaped, double-membraned phagophore, whose edges extend and fuse to become an autophagosome. A key event that marks the formation of phagophores is the conjugation of members of the ubiquitin-like ATG8 family, including the LC3 and GABARAP subfamilies, to phosphatidylethanolamine on precursor membranes. LC3/GABARAP remain on autophagosomes until after fusion with lysosomes.

The source of autophagosomes has been a key question in the field and many donor membranes have been proposed, including the endoplasmic reticulum (ER), ER/mitochondria contact sites (MAM), ER exit sites, recycling endosomes, Golgi and plasma membrane (Axe et al., 2008, Biazik et al., 2015, Dooley et al., 2014, Ge et al., 2013, Hamasaki et al., 2013, Hayashi-Nishino et al., 2009, Karanasios et al., 2016, Knaevelsrud et al., 2013b, Longatti et al., 2012, Puri et al., 2013, Ravikumar et al., 2010, Shibutani and Yoshimori, 2014, Yla-Anttila et al., 2009). One possibility is that autophagosomes are formed *de novo* by fusion of vesicles from various sources. Alternatively, they may form on a core platform that may receive inputs from secondary compartments. Thus, one needs to discriminate between any core platform on which autophagosomes form (as operationally defined by the membranes to which LC3 is conjugated) versus membranes/vesicles from different organelles that traffic to such sites bringing proteins and lipids required for autophagosome biogenesis. This platform is likely to be related to what was previously called “isolation membrane.” While the nature of the isolation membrane/autophagosome platform is still unclear, isolation membranes appear as membranes close to the rough ER and/or ER-mitochondria contact sites (MAM) (Axe et al., 2008, Hamasaki et al., 2013, Hayashi-Nishino et al., 2009, Kishi-Itakura et al., 2014, Yla-Anttila et al., 2009).

We previously described trafficking of mATG9 and ATG16L1 in different vesicles from the plasma membrane, which meet in recycling endosomes. The fusion of these mATG9- and ATG16L1-containing vesicles regulates subsequent LC3 lipidation and autophagosome formation (Puri et al., 2013). The interpretation of this and other related studies (Haobam et al., 2014, Knaevelsrud et al., 2013a, Longatti et al., 2012, Orsi et al., 2012, Szatmari et al., 2014) was that membranes from recycling endosomes traffic to sites of autophagosome biogenesis close to the ER (Hamasaki et al., 2013, Shibutani and Yoshimori, 2014, Tooze et al., 2014). Thus, while previous studies have implicated recycling endosomes as a membrane source for autophagosomes, they had not considered this organelle as the foundation structure on which autophagosomes form.

The sites of LC3 conjugation (and thus the platform membranes) are specified by ATG16L1 (Fujita et al., 2008b), which is recruited to the sites of autophagosome formation by interacting with WIPI2, a protein that associates with membranes enriched in phosphatidylinositol 3-phosphate (PI3P) (Dooley et al., 2014, Vicinanza et al., 2015). However, as these phosphoinositides are found in many sites in the cell, in addition to those where autophagosomes form, it is unlikely that they constitute the only signal for determining where WIPI2/ATG16L1 is recruited. Conventionally, phosphatidylinositides recruit proteins via coincident mechanisms in concert with other proteins (e.g., small GTPases; Carlton and Cullen, 2005). Thus, we hypothesized the existence of a protein on the isolation membrane that engages WIPI2 and targets the WIPI2-ATG16L1 complex.

Here we describe that WIPI2 is recruited to the platform of autophagosome formation by RAB11A, a core component of recycling endosomes (where we also detected newly formed PI3P in response to starvation). This enabled characterization of the RAB11A-positive membranes as a platform on which autophagosomes assemble.

## Results

### WIPI2 Interacts with RAB11A

**Figure 1:**
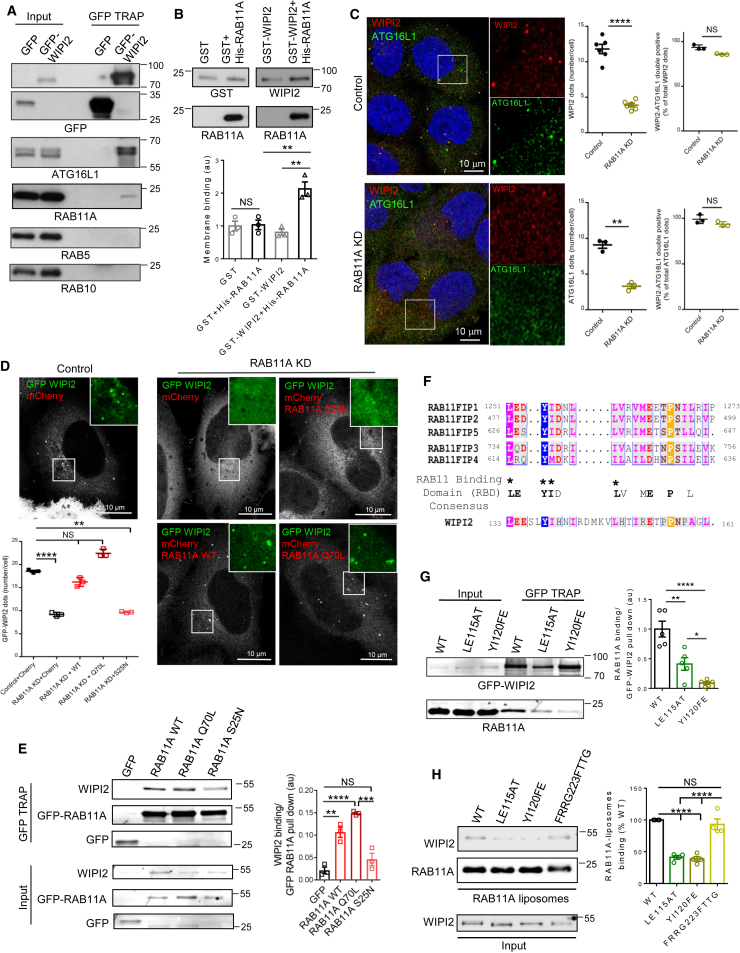
WIPI2 Interacts with RAB11A (A) GFP-WIPI2 and GFP were immuno-precipitated using GFP-TRAP on lysates from HeLa cells stably expressing GFP-WIPI2 or GFP-empty vector under starvation conditions (EBSS) for 2 hr; blots were probed as indicated. (B) Binding of GST-WIPI2 to RAB11A-liposomes was analyzed by probing for liposome-bound GST (GST antibody lanes 1–2) or GST-WIPI2 (WIPI2 antibody, lanes 3–4) (see STAR Methods). Data are means ± SEM, n = 3; One-way ANOVA with post hoc Tukey's test, ^∗∗^p < 0.01; NS, not significant. (C) HeLa cells treated with control or RAB11A siRNA, starved for 2 hr, and labeled for WIPI2 and ATG16L1. Quantification of WIPI2-, ATG16L1-single-positive structures (number/cell) is shown. WIPI2-ATG16L1 double-positive structures are expressed as percentage of total WIPI2 or ATG16L1 structures. Data are means ± SEM (n = 6 for WIPI2, n = 3 for ATG16L1, 50 cells per condition); two-tailed paired *t* test, ^∗∗^p < 0.01, ^∗∗∗∗^p < 0.0001; NS, not significant. (D) HeLa cells treated as in (A) transfected with GFP-WIPI2 in combination with mCherry-empty or mCherry-RAB11A WT or mutants. Quantification of WIPI2 structures/cell is shown. Data are means ± SEM (n = 3, 40 cells per condition); One-way ANOVA with post hoc Tukey's test, ^∗∗^p < 0.01, ^∗∗∗∗^p < 0.0001; NS, not significant. (E) HeLa cells transfected with GFP-RAB11A WT or mutants were starved for 1 hr and processed for GFP-TRAP. The amount of WIPI2 pull-down by RAB11A WT and mutants is shown. Data are means ± SEM (n = 3 independent experiments); one-way ANOVA with post hoc Tukey's test, ^∗∗^p < 0.01, ^∗∗∗^p < 0.001, ^∗∗∗∗^p < 0.0001; NS, not significant. (F) Alignment of amino acid sequence of the RAB11-binding domain (RBD) of RAB11FIPs with WIPI2 (isoform B, residues 133–161). Colored box, white character strict identity. Consensus sequence for RAB11 binding is shown; hydrophobic residues forming a RAB11-binding patch are marked with asterisks. (G) HeLa cells transfected with GFP-WIPI2 WT, GFP-WIPI2 LE115AT and GFP-WIPI2 YI120FE and starved for 2 hr were processed for GFP-TRAP as in (A). Data are means ± SEM, n = 5; one-way ANOVA, ^∗^p < 0.05, ^∗∗^p < 0.01, ^∗∗∗∗^p < 0.0001. (H) Binding of WT, LE115AT, YI120FE, and FRRG223FTTG WIPI2-FLAG recombinant proteins to RAB11A-containing liposomes was measured *in vitro* by liposome sedimentation assay (see STAR Methods). WIPI2 association with liposomes is shown. Data are means ± SEM, n = 4; one-way ANOVA with post hoc Tukey's test, ^∗∗∗∗^p < 0.0001; NS, not significant.

To identify new regulators of WIPI2 localization on forming autophagosomes, we used mass spectrometry and identified RAB11A as a putative WIPI2 interactor (Figure S1A). This interaction was confirmed by immunoblot, using GFP-TRAP on lysates from GFP-WIPI2 (Figure 1A) or GFP-RAB11A stable cell lines (Figure S1B). Consistent with previous reports (Dooley et al., 2014), ATG16L1 co-immunoprecipitated with WIPI2 (Figure 1A), validating our approach. The interaction of endogenous WIPI2 with RAB11A was confirmed in both basal and starvation conditions (Figure S1C) and *in vitro* using liposomes, where we mimicked RAB11A membrane-anchoring using His-tagged RAB11A and liposomes bearing Ni^2+^-NTA-modified lipids (Ni^2+^ binds His-tags on recombinant proteins) (Viaud et al., 2014) (Figure 1B). Sedimentation assays showed that the WIPI2-membrane association was highly dependent on the presence of RAB11A on liposomes (Figure 1B).

In living cells, WIPI2 localized at membranes positive for the recycling endosome markers RAB11A and RAB10, appearing in globular structures emanating from and in contiguity with RAB11A/RAB10 vesicles/tubules (Wang et al., 2016) (Figures S1D, S1E, and S1G). Importantly, the WIPI2 distribution was not affected by the constitutively active form of the early endosomal GTPase RAB5, RAB5Q79L, which is widely used to identify proteins trafficking through the early endosomes (Figures S1F and S1G), and RAB5 was not detected in GFP-WIPI2 pull-downs (Figure 1A).

When autophagy was induced (i.e., by nutrient starvation), WIPI2 appeared on discrete puncta labeled by ATG16L1, another marker for phagophores (Figure 1C). RAB11A silencing strongly inhibited the appearance of WIPI2 and ATG16L1 on discrete puncta, representing forming autophagosomes (Figures 1C and S1H), with the WIPI2-ATG16L1 colocalization on puncta remaining unchanged (Figure 1C). WIPI2 re-appeared on forming autophagosomes when RAB11A was transfected back into RAB11A siRNA-treated cells (Figure 1D). While the RAB11A GTPase-deficient mutant, RAB11AQ70L, rescued WIPI2 and ATG16L1 puncta (Figures 1D and S1I) and pulled down WIPI2 (Figure 1E), the RAB11A GTP binding-defective mutant, RAB11AS25N, failed to rescue WIPI2 and ATG16L1 puncta (Figures 1D and S1I) and to interact with WIPI2 (Figure 1E). This allowed us to conclude that RAB11A contributes to autophagosome assembly mainly when in its active state.

Silencing of either RAB11A or WIPI2 inhibited autophagosome biogenesis, compatible with a common mechanism of action; when lysosomal degradation is inhibited with Bafilomycin A1, any changes in LC3 vesicles/LC3-II should reflect altered biogenesis (Figure S1J). We further confirmed RAB11A as a positive regulator of autophagosome biogenesis by measuring LC3-II synthesis by pulse-labeling newly synthesized LC3 (Figure S1K). These data are consistent with the inhibition of LC3 lipidation previously observed with a RAB11 dominant-negative mutant and with silencing of WIPI2 (Longatti et al., 2012, Mauthe et al., 2011, Polson et al., 2010). Depletion of either RAB11A or WIPI2 decreased LC3 vesicle numbers (autophagosomes) (Figures S1L and S1M) and increased the percentages of cells with aggregates of mutant huntingtin exon 1 (Figures S1N and S1O), a well-validated autophagy substrate (Narain et al., 1999). Supporting the idea that RAB11A in its active state plays a role in autophagy, only the RAB11A WT and the constitutively active mutant, RAB11AQ70L (but not the inactive mutant, RAB11AS25N), rescued LC3 vesicle numbers in RAB11A-depleted cells (Figure S1P).

### WIPI2 Binds RAB11A via a Conserved RAB11-Binding Sequence

Alignment of the amino acid sequence of human WIPI2 with the RAB11-binding domains (RBD) of the RAB11FIPs (RAB11 family of interacting proteins) (Prekeris et al., 2001) revealed a putative RBD domain in the WIPI2 sequence (Figure 1F). Some of the putative residues required for RAB11 binding were conserved in other members of the WIPI family and in their yeast counterparts (Figure S1Q). Consistent with a putative RBD in the WIPI1 sequence, we found that WIPI1 also pulled down RAB11A (Figure S1Q). Substitutions of residues in the strictly conserved LE and YI motifs in WIPI2 resulted in a significant reduction of RAB11A binding (Figures 1G, 1H, and S1R), with the YI120FE mutant having a more dramatic effect than the LE115AT mutant. These effects correlate with residues in RAB11 FIPs required for RAB11 binding (Prekeris et al., 2001).

**Figure 2:**
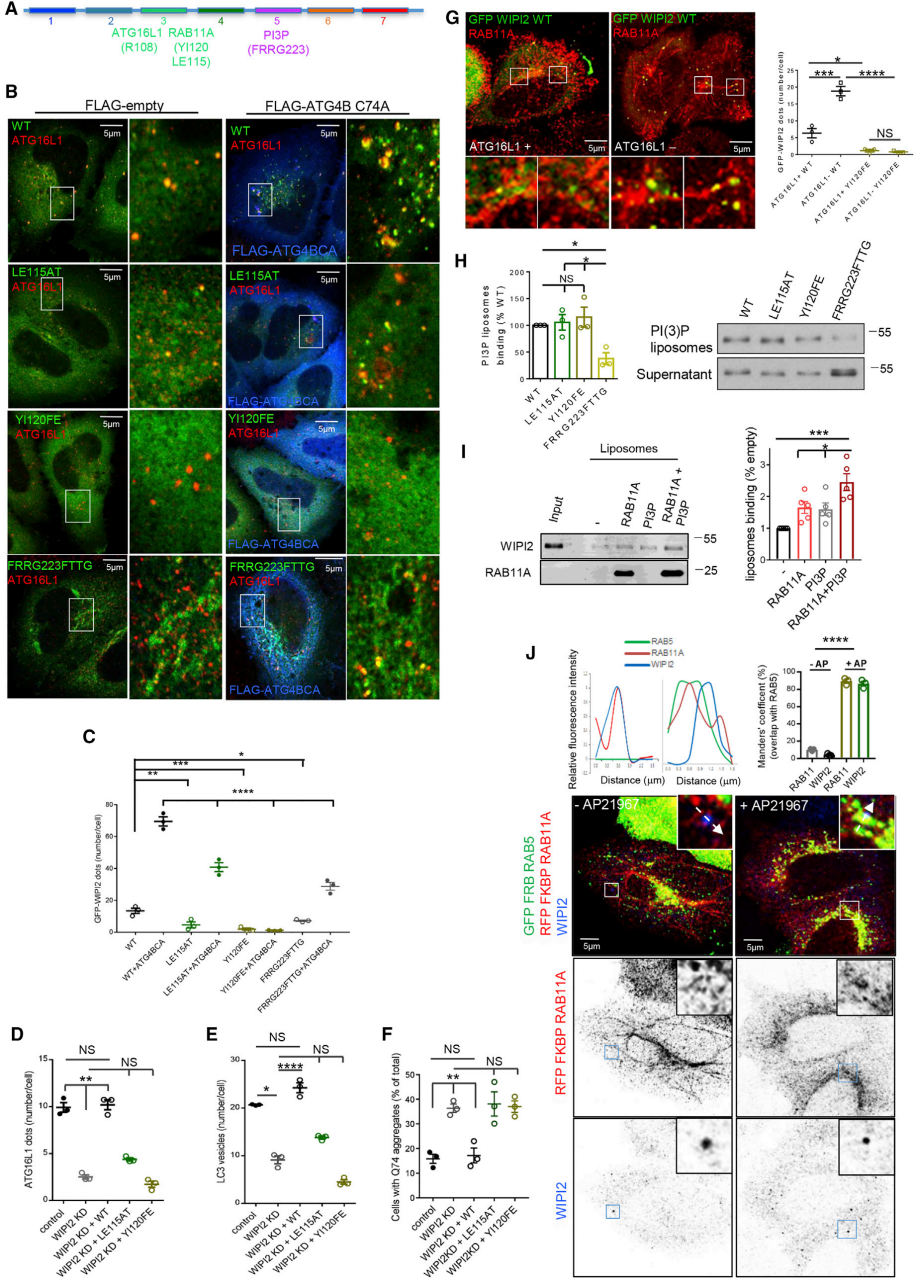
WIPI2 Mutants Defective in RAB11A Binding Fail to Localize on Autophagosomes and to Sustain Autophagosome Formation (A) Representation of the human WIPI2 protein harboring 7 WD repeats. (B and C) HeLa cells were transiently transfected with GFP-WIPI2 WT and mutants in combination with FLAG or FLAG-ATG4BC74A, starved for 2 hr, and labeled for FLAG and ATG16L1. The number of WT or mutant WIPI2 dots is shown. Data are means ± SEM, n = 3, 50 cells per condition; one-way ANOVA with post hoc Tukey's test, ^∗^p < 0.05, ^∗∗^p < 0.01, ^∗∗∗^p < 0.001, ^∗∗∗∗^p < 0.0001. (D–F) HeLa cells transfected with control or WIPI2 siRNA were transfected with GFP-empty, or GFP-WIPI2 WT and mutants, incubated for 2 hr (D) or 4 hr (E) in EBSS, and labeled for ATG16L1 (D) or LC3 (E). (D-E) ATG16L1 or LC3 vesicles number/cell are shown. In (F), WIPI2-silenced cells were transfected with GFP-empty or GFP-WIPI2 WT and mutants in combination with HA-tagged Htt-Q74 and processed for immunofluorescence. Number of cells with Htt-Q74 aggregates is shown. Data are means ± SEM, n = 3, 40 cells (D and E) or 800 cells (F) per condition; one-way ANOVA with post hoc Tukey's test, ^∗^p < 0.05, ^∗∗^p < 0.01, ^∗∗∗∗^p < 0.0001; NS, not significant. (G) CRISPR/Cas9 ATG16L1 knockout and control cells were transfected with GFP-WIPI2 WT, starved for 2 hr, and labeled for RAB11A. WIPI2 puncta/cell are shown. Data are means ± SEM, n = 3, 25 cells per condition; one-way ANOVA with post hoc Tukey's test, ^∗^p < 0.05, ^∗∗∗^p < 0.001, ^∗∗∗∗^p < 0.0001; NS, not significant. Also see Video S1. (H) Binding of purified WIPI2-FLAG proteins (WT and mutants) was measured by liposome sedimentation assay (see STAR Methods). WIPI2 binding to PI3P-liposomes is shown. Data are means ± SEM, n = 3; one-way ANOVA with post hoc Tukey's test, ^∗^p < 0.05; NS, not significant. (I) Liposomes carrying RAB11A, PI3P or combination of both were tested for the ability to recruit WIPI2 by liposome sedimentation assay (see STAR Methods). WIPI2 membrane binding is shown. Data are means ± SEM, n = 5; one-way ANOVA with post hoc Tukey's test, ^∗^p < 0.05, ^∗∗∗^p < 0.001. (J) HeLa cells transiently transfected with GFP-FRB-RAB5A and RFP-FKBP-RAB11A were treated with 250 nM AP21967 for 30 min in full-media and stained for WIPI2 and anti-RFP. The graphs on the left show the relative fluorescence intensity of the area under the arrows; the histogram on the right shows quantification of RAB11A/WIPI2 translocation to early endosomes by addition of AP21967. Data are means ± SEM, n = 3; one-way ANOVA with post hoc Tukey's test, ^∗∗∗∗^p < 0.0001.

WIPI2 mutants defective in RAB11A binding rarely (LE115AT) or never (YI120FE) formed WIPI2-positive-structures (phagophores), compared with the wild-type protein (Figures 2A–2C). These defects were enhanced when WIPI2 localization was assessed in the presence of a proteolytic activity-deficient mutant of ATG4B (ATG4BC74A) that prevents autophagosome completion (Fujita et al., 2008a) (Figures 2B and 2C), suggesting a crucial role for RAB11A in WIPI2 recruitment at the initiation membrane. Consistent with the accepted role for WIPI2 to recruit the ATG16L1 complex to nascent phagophores (Dooley et al., 2014), the numbers of ATG16L1 puncta (Figure 2D) and autophagosomes (Figure 2E) were dramatically reduced in WIPI2 small interfering RNA (siRNA)-treated cells and the rescue of these knockdown phenotypes was impaired by the LE115AT and YI120FE mutations compared with wild-type WIPI2 (Figures 2D and 2E). WIPI2 overexpression increased autophagy and degradation of conventional cytoplasmic autophagic substrates, such as p62 (SQSTM1) and mutant huntingtin exon 1; all these effects were compromised by the LE115AT and YI120FE mutants (Figures S2A and S2B), consistent with a defect in autophagy. Similarly, unlike wild-type WIPI2, these mutants failed to decrease the levels of mutant huntingtin aggregates that were elevated in WIPI2-depleted cells (Figure 2F).

In cells lacking ATG16L1 (Bento et al., 2016), WIPI2 pulled down more RAB11A (Figure S2C) and localized more on puncta, mostly on RAB11A-positive membranes (Figure 2G, Video S1). These effects were lost when the YI120FE WIPI2 mutant was used (Figures 2G and S2C). Likewise, a WIPI2 mutant defective in ATG16L1 binding (R108E previously described in Dooley et al., 2014) (Figure S2D), or ATG16L1 silencing by siRNA (Figure S2E), phenocopied the increased amount of RAB11A pulled down by WIPI2. Thus, RAB11A binding to WIPI2 does not require ATG16L1 (Figures 2A, 2G, and S2C–S2E). Consistent with the ATG16L1-WIPI2 interaction (Dooley et al., 2014 and Figure 1A), ATG16L1 was immunoprecipitated by RAB11A (Figure S2F). This was more pronounced when cells were exposed to the autophagy-inducing stimulus of starvation, while it was reduced by WIPI2 knockdown (Figure S2F). RAB11A knockdown appeared to decrease the ATG16L1-WIPI2 interaction (Figure S2E). The YI120FE WIPI2 mutation compromised its interaction with ATG16L1 (Figure S2D), partially explaining the defective autophagosome formation in RAB11A-silenced cells (Figures 1C, 1D, S1H, S1I, and S1P) and the reduction in ATG16L1 puncta in cells expressing WIPI2 RBD-mutants (Figure 2D). WIPI2 RBD-mutants were still able to bind ATG16L1 *in vitro* (Figure S2G), but the WIPI2 mutants localized *in vivo* in different compartments from ATG16L1 (Figure S2H), which likely explains why WIPI2 RBD-mutants showed reduced binding to ATG16L1 in cells (Figure S2D). These data suggest that WIPI2 functions between RAB11A and ATG16L1; while ATG16L1 depletion increases the amounts of RAB11A-WIPI2 that interact (Figures S2C and S2E), loss of WIPI2 decreases the RAB11A/ATG16L1 interaction (Figure S2F).

The only mutant of WIPI2 so far described that fails to localize at autophagosomes is FRRG223FTTG, which is unable to bind to PI3P (Dooley et al., 2014). Surprisingly, WIPI2 FRRG223FTTG had no obvious impairment in its binding to RAB11A (Figures 1H and S2D). On the other hand, the RAB11-binding-defective WIPI2 (LE115AT and YI120FE) mutants retained the ability to bind PI3P (Figure 2H), suggesting that the binding modules for RAB11A and PI3P are distinct on WIPI2 (Figure 2A). However, WIPI2 mutants defective in RAB11A-binding, similar to what reported for WIPI2 mutant defective in PI3P-binding (Dooley et al., 2014 and Figure S2H), failed to redistribute to autophagosomes (Figures 2B, 2C, and S2H), suggesting that PI3P is necessary but not sufficient for WIPI2 recruitment. Importantly, the PI3P-binding-defective (FRRG223FTTG) mutant, despite binding to and co-localizing with RAB11A in a perinuclear compartment (in both basal and starvation conditions), formed significantly fewer WIPI2-positive structures (Figure S2H), even when autophagosome precursors were forced to accumulate by exogenous expression of the ATG4BC74A mutant (Figures 2B and 2C). Conversely, the GFP WIPI2 YI120FE mutant remained completely diffuse in both basal and starvation conditions (Figure S2H), and, unlike the FRRG223FTTG mutant, did not show any increase in vesicles even when autophagosome closure was blocked by ATG4BC74A (Figures 2B and 2C).

While the presence of either RAB11A or PI3P on liposomes was sufficient to stabilize WIPI2 on membranes, a combination of RAB11A and PI3P on the same liposome synergistically enhanced WIPI2 membrane binding compared with control liposomes and to liposomes carrying only one factor (Figure 2I). To test whether this is also the case in live cells, artificial localization of RAB11A to a PI3P-enriched compartment (i.e., early endosomes) was performed in nutrient-rich media using the FRB:FKBP chemically induced heterodimerization system controlled by the non-immunosuppressive rapamycin analog AP21967 (Hammond et al., 2014, van Bergeijk et al., 2015). In untreated cells, FRB-RAB5 and FKBP-RAB11A localized to distinct compartments (early and recycling endosomes, respectively) and endogenous WIPI2 associated with the RAB11 compartment (Figure 2J). The addition of AP21967 resulted in the efficient translocation of FKBP-RAB11A to RAB5-early endosomes and in an increased number of WIPI2 puncta on RAB11/RAB5-positive endosomes (Figure 2J). AP21967 treatment did not cause obvious re-localization of WIPI2 on early endosomes when a RAB11A plasmid lacking the FKBP tag was used (Figure S2I). Our observations are consistent with the coincident detection model where PI3P and RAB11A work in concert on WIPI2 to define the site of autophagosome biogenesis and sustain autophagosome formation on RAB11A-positive recycling endosomes under starvation.

### ATG Proteins Localize Mostly on RAB11A-Positive Compartment

**Figure 3:**
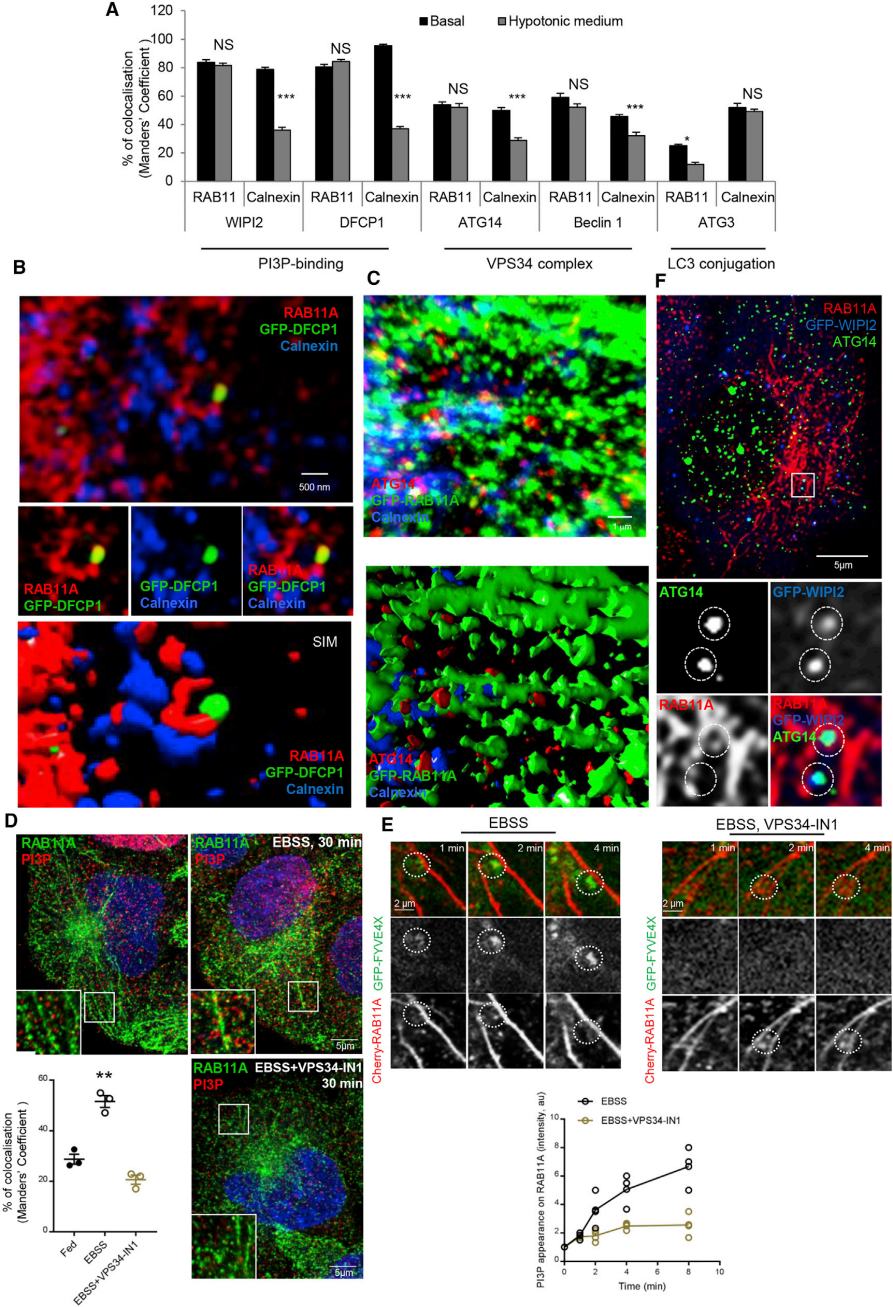
Autophagic Proteins Are in RAB11A Compartments (A) Colocalization of different autophagic proteins with RAB11A or calnexin (MAM) in basal or under hypotonic medium treatment is shown. Data are means ± SEM, n = 3, 20 cells per condition; two-tailed paired *t* test; ^∗^p < 0.05; ^∗∗∗^p < 0.001; NS, not significant. Relevant images in Figure S3. (B) HeLa transfected with GFP-DFCP1 were labeled for RAB11A and calnexin and visualized on Elyra (Zeiss) superresolution microscope. (C) GFP-RAB11A HeLa labeled for ATG14 and calnexin and processed for 3D z-stack reconstruction using Volocity software. (D) HeLa cells treated with VPS34-IN1 were stained for RAB11A and PI3P and the overlap between PI3P/RAB11A was measured. Data are means ± SEM, n = 3, 35 cells per condition; one-way ANOVA, ^∗∗^p < 0.01. (E) HeLa cells transfected with mCherry-RAB11A and GFP-FYVE4X were recorded under starvation in presence or absence of VPS34-IN1. Corresponding regions of the GFP-FYVE4X and Cherry-RAB11A channels are shown with dashed circles. (F) HeLa cells transfected with GFP-WIPI2, starved for 2 hr, and labeled for RAB11A and ATG14. Corresponding regions of the different channels are shown with dashed circles.

It is well accepted that the key signal for autophagosome initiation is a local increase of PI3P on a membrane that is then defined “isolation membrane” as a result of recruitment of PI3P-binding autophagic proteins (Nascimbeni et al., 2017). On the other hand, an extensive literature shows “isolation membrane” arising between two cisternae of rough ER and/or in proximity of ER-mitochondria contact sites (MAM) (Axe et al., 2008, Hamasaki et al., 2013, Hayashi-Nishino et al., 2009, Kishi-Itakura et al., 2014, Uemura et al., 2014, Yla-Anttila et al., 2009). To examine if the RAB11A-positive membranes could represent the extensively described “isolation membrane” enriched in PI3P and in proximity of MAM (Biazik et al., 2015, Hayashi-Nishino et al., 2009, Karanasios et al., 2016), we investigated diverse PI3P-connected ATG proteins associated with the early stages of autophagosome formation to establish if they localized to RAB11A-positive membranes or to the MAM (calnexin-positive) (Figures 3A and S3). As MAM and recycling endosomes are in very close proximity, colocalization studies were performed under acute microtubule depolymerization treatments (5 min hypotonic medium) that allow separation of recycling endosome and MAM compartments (Brinkley et al., 1980). We used this acute approach to minimize the widespread effects of microtubule disruption. The specific association of WIPI2 with RAB11A-positive membranes was confirmed (Figures 3A and S3A), validating the WIPI2-RAB11A interaction described above (Figures 1, S1, and S2). DFCP1 (Double FYVE domain containing protein 1) that binds PI3P and identifies the omegasome, was also located in the recycling endosomes by superresolution structured illumination microscopy (SIM) (Figure 3B). Interestingly, previous reports localized GFP-DFCP1 on tubular or vesicular elements adjacent to the IM (isolation membrane) rims (Uemura et al., 2014). Our data suggest that these tubular structures are the RAB11A-positive compartments close to the MAM (Figures 3A, 3B, and S3B).

In addition to PI3P-binding proteins, core components of the autophagic VPS34 complex, ATG14 and Beclin1, responsible for the PI3P at the nascent autophagosomes (Axe et al., 2008) also localized on RAB11A-positive membranes (Figures 3A, 3C, S3C, and S3D). As a consequence of components of VPS34-ATG14 complex localizing on RAB11A-positive membranes, we assessed if PI3P increased on these membranes in response to starvation, which we confirmed (Figures 3D and 3E). Treatment with VPS34 inhibitor (VPS34-IN1) (Bago et al., 2014) made the PI3P signal on RAB11A-positive membranes disappear (Figures 3D and 3E). This correlates with the localization of WIPI2 and ATG14 on the same autophagic structures on recycling endosomes (Figure 3F) and with the partial dispersion of WIPI2 in ATG14-depleted cells (Figure S3E). It is worth noting that the enrichment of PI3P on RAB11A-positive membranes under starvation provides the second important signal on these membranes, in addition to RAB11A, for WIPI2 stabilization on nascent phagophores.

While microtubule depolymerization reduced the association of WIPI2, DFCP1, ATG14, and Beclin 1 with the MAM marker calnexin (Figures 3A, S3A, and S3B), this was not the case of ATG3 (Figures 3A and S3F), the E2-like enzyme that promotes LC3 lipidation during phagophore expansion. This appears to be consistent with the reported enrichment of ATG3 in the ER-Golgi intermediate compartment (Ge et al., 2013) and the involvement of other compartments (ER/MAM) in the later stages of autophagosome formation.

### LC3 Is Associated with the RAB11A Compartment

**Figure 4:**
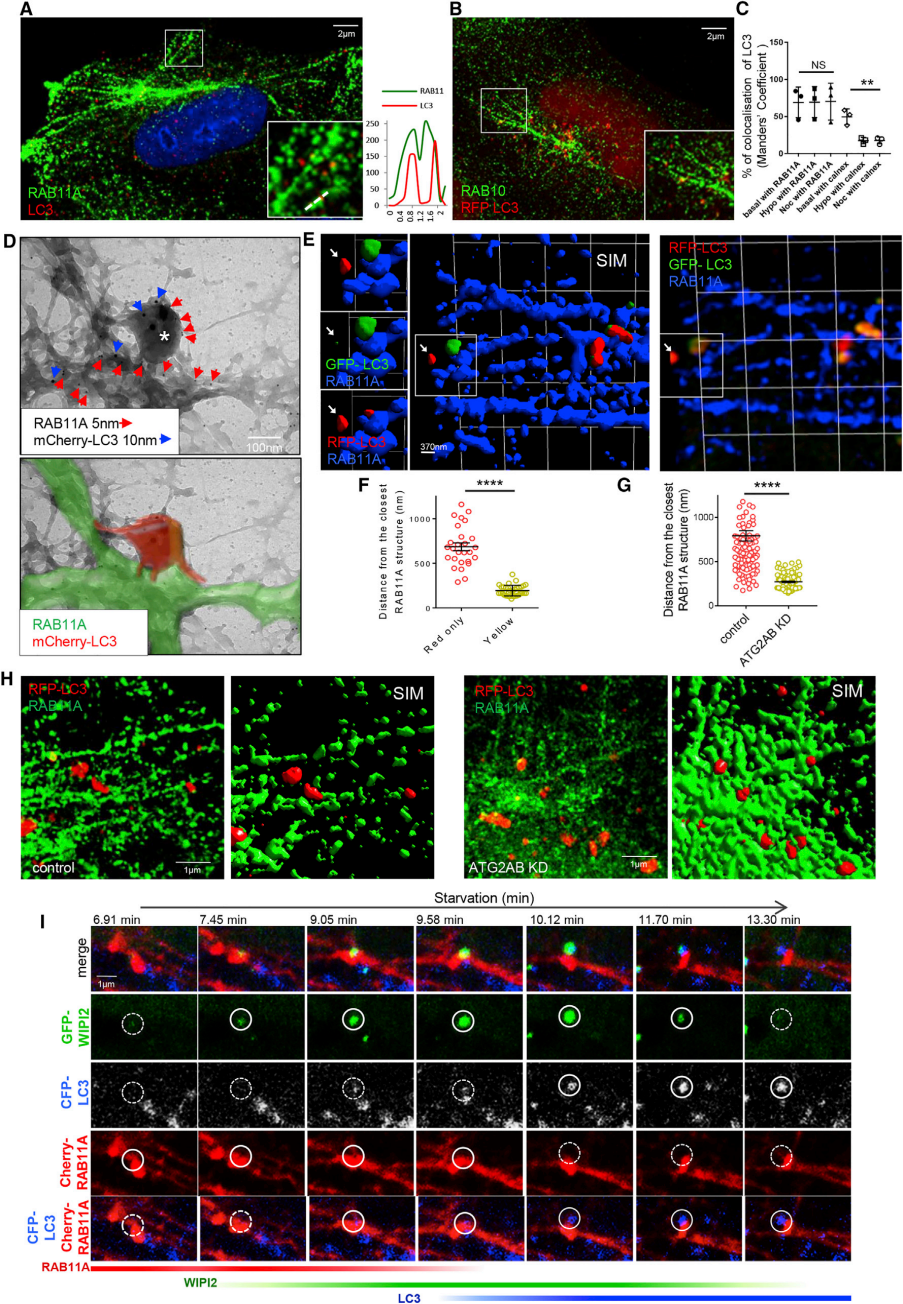
LC3 Is in RAB11A Compartments (A) HeLa cells labeled for RAB11A and LC3. The graphs show the relative fluorescence intensity of the area under the arrow. See also Video S2. (B) HeLa cells transfected with RFP-LC3 and labeled for RAB10. (C) Colocalization of RFP-LC3 with RAB11A or calnexin in cells treated or not with hypotonic medium or nocodazole. Data are means ± SEM, n = 3, 20 cells per condition; two-tailed paired t test, ^∗∗^p < 0.01; NS, not significant. Relevant images in Figure S4I. (D) HeLa cells transfected with PAM-mCherry-LC3 and processed for whole-mount immunoelectron microscopy (see STAR Methods). The asterisk indicates the LC3-positive structure. The edges of the recycling endosomes (green) and the autophagic structure (red) are highlighted below. (E and F) HeLa cells transfected with RFP-GFP tandem fluorescent-tagged LC3 were labeled for the RAB11A and processed for superresolution microscopy. The arrow indicates an acidified red-only autophagosome separate from the RAB11A compartment. In (F), the quantification of the distance (nm) of LC3 dots (red only or yellow) from the closest RAB11A tubule was assessed using Volocity 6.3 Software. Data are means ± SEM (30 structures per condition); two-tailed paired t test, ^∗∗∗∗^p < 0.0001. (G and H) HeLa cells treated with ATG2A/B siRNA, transfected with RFP-LC3 and labeled for RAB11A were processed for superresolution microscopy and the distance of LC3 dots from the closest RAB11A tubule quantified (G) as in (F). Data are means ± SEM, 130 structures per condition; two-tailed paired t test, ^∗∗∗∗^p < 0.0001. (I) HeLa cells transfected with GFP-WIPI2, mCherry-RAB11A and CFP-LC3 were imaged for 30 min (see also Video S3). The area delimited by a circle shows a new autophagosome forming on recycling endosome. Dashed line circles were used in time frames where the fluorescence signal disappears.

LC3-positive puncta decorated the RAB11A-positive tubules (Figure 4A, Video S2). The LC3 vesicles also associated with RAB10, another recycling endosome marker (Figure 4B). The specificity of the LC3 vesicles was indicated by their colocalization with RAB11A in wild-type mouse embryonic fibroblasts (MEFs), but its absence in autophagy-incompetent ATG16L1-null MEFs (Cadwell et al., 2008) (Figure S4A) or in autophagy-incompetent cells (ULK1- and FIP200-depleted cells) where the LC3-RAB11A colocalization is decreased (Figures S4B–S4D). Similar LC3 localization on the RAB11 compartment was also observed in other cell lines, such as neuroblastoma cells (SHSY5Y) or mouse primary cortical neurons (Figures S4E and S4F).

Similarly, vesicles containing the ATG8 family members GABARAP and GABARAPL1 involved in autophagosome formation (Kabeya et al., 2004, Weidberg et al., 2010) localized on RAB11A tubules (Figures S4G and S4H).

As we described above with other ATGs (Figure 3A), the association of LC3 with RAB11A appeared resistant to two microtubule depolymerization treatments (nocodazole or 5 min hypotonic medium) (Figures 4C and S4I). Conversely, the LC3-MAM colocalization/association was significantly reduced by microtubule depolymerization (Figures 4C and S4I).

We validated these confocal microscopy observations using immunoelectron microscopy in whole-mounted cells, an approach used previously to examine transferrin receptor-containing endosomes/recycling endosomes in non-sectioned cells (Stoorvogel et al., 1996). With this approach, these endosomes appear as separate discontinuous tubular-vesicular entities. Saponin treatment, which removes cytoplasm, makes the cells electron-lucent and enables colloidal gold labeling of the cytoplasmic tails of integral membrane proteins in endosomes, or proteins associated with the cytoplasmic face of endosomes (Stoorvogel et al., 1996). Using this method, we observed that the RAB11A compartment appeared as tubular structures (highlighted in green) (as previously shown), from which LC3-positive structures emerge (highlighted in red) (Figure 4D).

In order to assess if the LC3-positive structures associated with RAB11A-positive membranes were autophagosomes/phagophores or autolysosomes, we transfected cells with tandem tagged mRFP-GFP-LC3 in order to discriminate between autophagosomes/phagophores and autolysosomes, since the former emit both RFP and GFP signals, whereas the acidic pH in lysosomes denatures GFP so that only RFP fluorescence is observed (Kimura et al., 2007). Using superresolution SIM (Huang et al., 2009, Ingaramo et al., 2014), we quantified the RFP + GFP (yellow) or RFP-only (red only) structures associated with RAB11A, and observed that most were both red and green (i.e., autophagosomes/phagophores), while the RFP-only structures (autolysosomes) were mostly detached from the RAB11A compartment (Figures 4E and 4F). When we depleted cells of ATG2A/B in order to impair autophagosome closure and completion (Tamura et al., 2017, Velikkakath et al., 2012), LC3 vesicles (nascent autophagosomes) aberrantly decorated the RAB11A-positive membranes, consistent with them not being able to leave the compartment (Figures 4G and 4H), supporting the idea that autophagosome formation occurs on these membranes. The likelihood that the LC3-positive structures associated with RAB11A-positive membranes were nascent autophagosomes was supported by live cell imaging experiments, where WIPI2 appeared on RAB11A-positive membranes upon starvation, and marked the sites to which LC3 would subsequently become associated (Figure 4I and Video S3). Interestingly, the WIPI2 signal is lost from the nascent autophagosomes after the LC3 signal is established, and the process is dynamic (with the WIPI2 residence on these structures being only about 5 min) (Figure 4I and Video S3).

### TfR Is Recruited on LC3-Positive Structures

**Figure 5:**
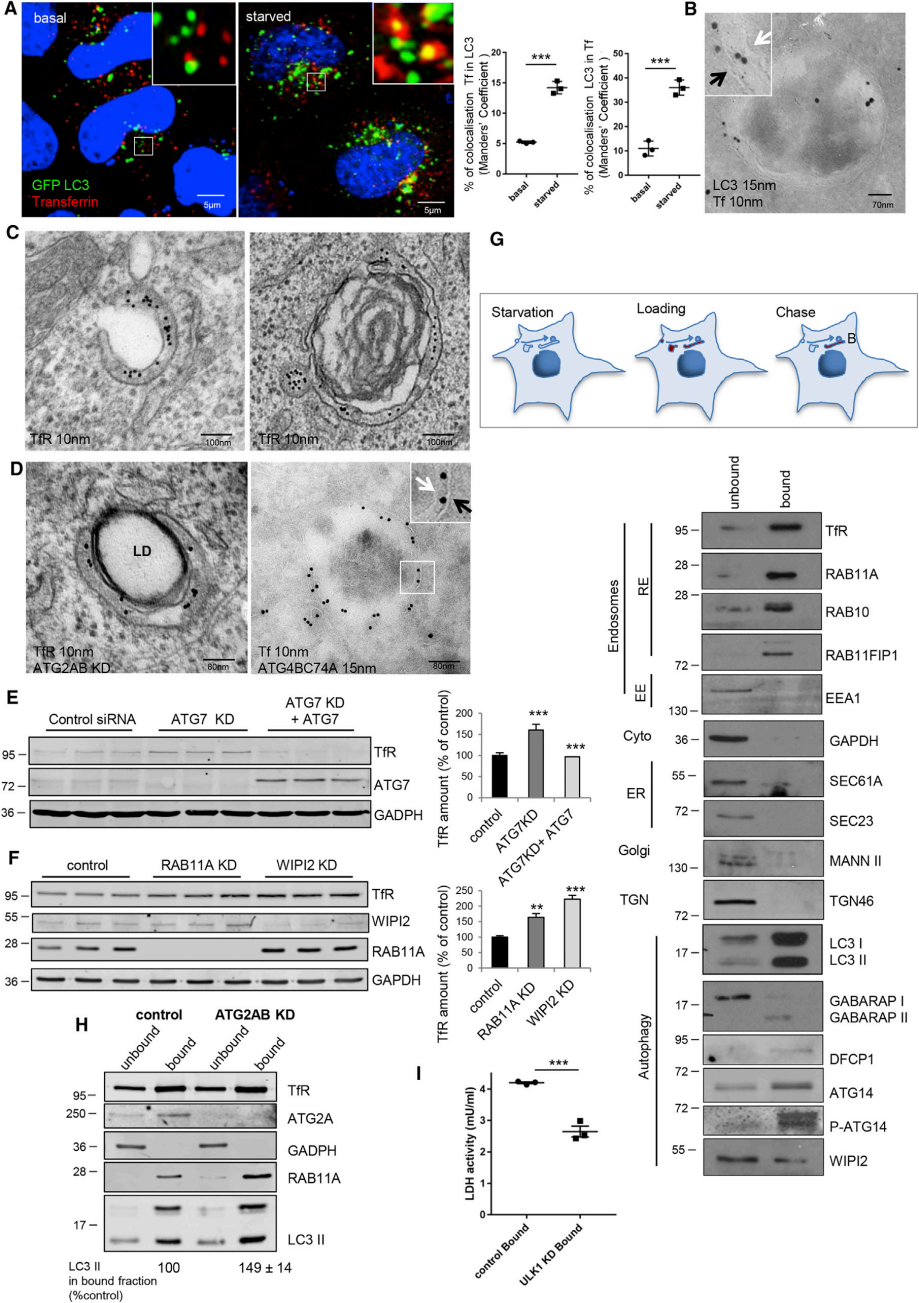
TfR Is Recruited to LC3 Vesicles in Starvation Conditions (A) HeLa cells transfected with GFP-LC3, were loaded with transferrin Alexa 555 for 1 hr in Hank’s balanced salt solution or full medium and chased for 15 min. The Tf/LC3 and LC3/Tf signal overlaps are shown. Data are means ± SEM, n = 3; two-tailed paired t test, ^∗∗∗^p < 0.001. (B) HeLa cells treated as in (A) were processed for immunogold labeling on cryosections. Arrows specify autophagosome double membranes, black outer, white inner. (C) HeLa cells starved for 1 hr and loaded with anti-TfR antibody were processed for pre-embedding electron microscopy. (D) HeLa cells treated with ATG2A/B siRNA (left) were processed as in (C). HeLa cells transfected for Flag-ATG4BC74A were loaded with Tf and processed as in (B). LD, lipid droplet. Arrows specify autophagosome double membranes as in (B). (E) HeLa cells treated with control or ATG7 siRNA and/or transfected with ATG7 were processed for immunoblotting and the TfR levels measured. Data are means ± SD, n = 3; two-tailed paired t test, ^∗∗∗^p < 0.001. (F) HeLa cells treated with control or RAB11A or WIPI2 siRNAs were processed as in (E). Data are means ± SD, n = 3; two-tailed paired t test, ^∗∗^p < 0.01, ^∗∗∗^p < 0.001. (G) HeLa cells starved for 1 hr, loaded with Ferrofluid-Tf Alexa 488 for 1 hr and chased for 15 min in starvation medium. The cells were then fragmented and the membranes containing Ferrofluid-Tf488 (bound) or not containing Ferrofluid-Tf488 (unbound) were separated and processed for immunoblotting (see Figure S5G). (H) HeLa cells transfected with control or ATG2A/B siRNA were processed as in (G). The amount of LC3II in bound fraction in ATG2 knockdown condition is expressed as percentage of control. Data are means ± SEM, n = 3. (I) HeLa cells treated with control or ULK1 siRNA and processed as in (G). The LDH activity was measured in the bound fraction. Data are means ± SEM, n = 3; two-tailed paired t test, ^∗∗∗^p < 0.001.

Transferrin and its cognate receptor are internalized by clathrin-mediated endocytosis and recycled back to the plasma membrane through early endosomes, or transferred to recycling endosomes for slower recycling back to the plasma membrane (Maxfield and McGraw, 2004). Transferrin and its receptor were previously described to localize to forming autophagosomes (Longatti et al., 2012), consistent with a model where recycling endosomes are the platforms for autophagosome biogenesis. After transferrin was internalized, it appeared mostly in early endosomes (EEA1) in nutrient-replete cells (when autophagy is not induced) and predominantly in recycling endosomes (RAB11A) in starvation conditions (when autophagy is induced) (Figure S5A). Internalized transferrin/transferrin receptor co-localized with LC3 and also with early autophagic markers (DFCP1, ATG14, WIPI2, and ATG16L1) in autophagy-inducing conditions (amino acid starvation) (Sarkar et al., 2007) (Figures 5A and S5B–S5D). Electron microscopy analysis confirmed that transferrin or the transferrin receptor localized between the inner and outer autophagosome membranes identified by LC3 labeling (Figures 5B and 5C). Strikingly, the localization inside the double membrane was evident in cells where autophagosome closure was impaired, such as in cells silenced for ATG2A/B or overexpressing the ATG4BC74A mutant (Figure 5D). Importantly, the ultrastructural analysis strengthens our findings that the autophagosomes/phagophores labeled by recycling endosome markers are generated by canonical autophagy (double membrane) and are not the single-membrane structures associated with non-canonical autophagy pathways, such as LAP (LC3-associated phagocytosis) or endocytic equivalents of LAP (Galluzzi et al., 2017). When the core autophagy protein ATG7 was silenced, we observed an accumulation of TfR, which was normalized by re-expressing exogenous ATG7 in the ATG7-depleted cells (Figure 5E). When autophagy was induced by SMER28, transferrin receptor co-localized with LC3 (in a RAB11A-positive compartment) and the level of TfR was substantially reduced (Figures S5E and S5F). Conversely, RAB11A or WIPI2 knockdowns caused the accumulation of this receptor (Figure 5F), suggesting that a new route for autophagic degradation occurs with TfR, where the substrate is embedded in the recycling endosomes/autophagosome membranes, as opposed to conventional substrates, which are sequestered within the inner membrane.

We exploited the fact that transferrin accumulates in recycling endosomes in response to starvation in order to aid the isolation of recycling endosome membranes. Transferrin Alexa 488 was conjugated with EMG 508 Ferrofluid, and then loaded and allowed to be internalized into cells in starvation conditions (Li et al., 2005). After the transferrin-Ferrofluid conjugates were delivered to the recycling endosome, cells were mechanically disrupted, the Ferrofluid-Tf-Alexa488-containing membranes were isolated using a magnet and then analyzed by western blot (Figures S5G and S5H). Using this approach, we confirmed that in starvation conditions, transferrin is recruited mostly on recycling endosomes (Figure S5I), as we observed with microscopy (Figure S5A). The Ferrofluid-Tf-Alexa488-containing membranes (Bound) were enriched in recycling endosome markers, such as transferrin receptor (TfR), RAB11A, the RAB11-binding protein RAB11FIP1, and RAB10, and not in early endosomes markers such as EEA1 (Figure 5G). In the unbound (U) membranes, we found ER and Golgi markers, such as SEC23, SEC61A, mannosidase II, and TGN46 (Figure 5G). WIPI2, an essential protein at the nascent autophagosome, was seen in the bound and unbound fractions (Figure 5G). Note that the pool of WIPI2 found in the unbound fraction is the cytosolic pool (Figure S5J), excluding the recruitment on other membrane compartments. Interestingly ATG14, a crucial component of the autophagic VPS34 complex at the nascent autophagosome, localized in both fractions but the phospho-ATG14 (that correlates with autophagosome formation) (Park et al., 2016) localized only to the bound membranes (Figure 5G). Importantly, lipidated/autophagosome-associated LC3 (LC3-II) and GABARAP (GABARAP-II) were highly enriched in the bound fraction as was the omegasome (autophagosome precursor) marker, DFCP1 (Figure 5G). Notably, similar results were obtained in SHSY5Y neuroblastoma cells (Figure S5K). In the absence of ATG2A/B, when autophagosome maturation is impaired, LC3 accumulated in the bound fraction, validating the idea that autophagosomes develop on recycling endosomes until their completion (Figure 5H). Note that ATG2A is enriched in bound fraction/recycling endosomes in control cells (Figure 5H).

When the critical autophagy protein ULK1 was depleted, we observed that the ability of the Ferrofluid-Tf-bound membranes to sequester the cytoplasmic enzyme, lactate dehydrogenase (LDH, a known autophagy substrate; Tsuboyama et al., 2016), was strongly impaired, suggesting that recycling endosomes are able to sequester cytoplasmic proteins in autophagy-dependent manner (Figure 5I).

### Autophagic Substrates Are Engulfed by RAB11A-Positive Compartment

**Figure 6:**
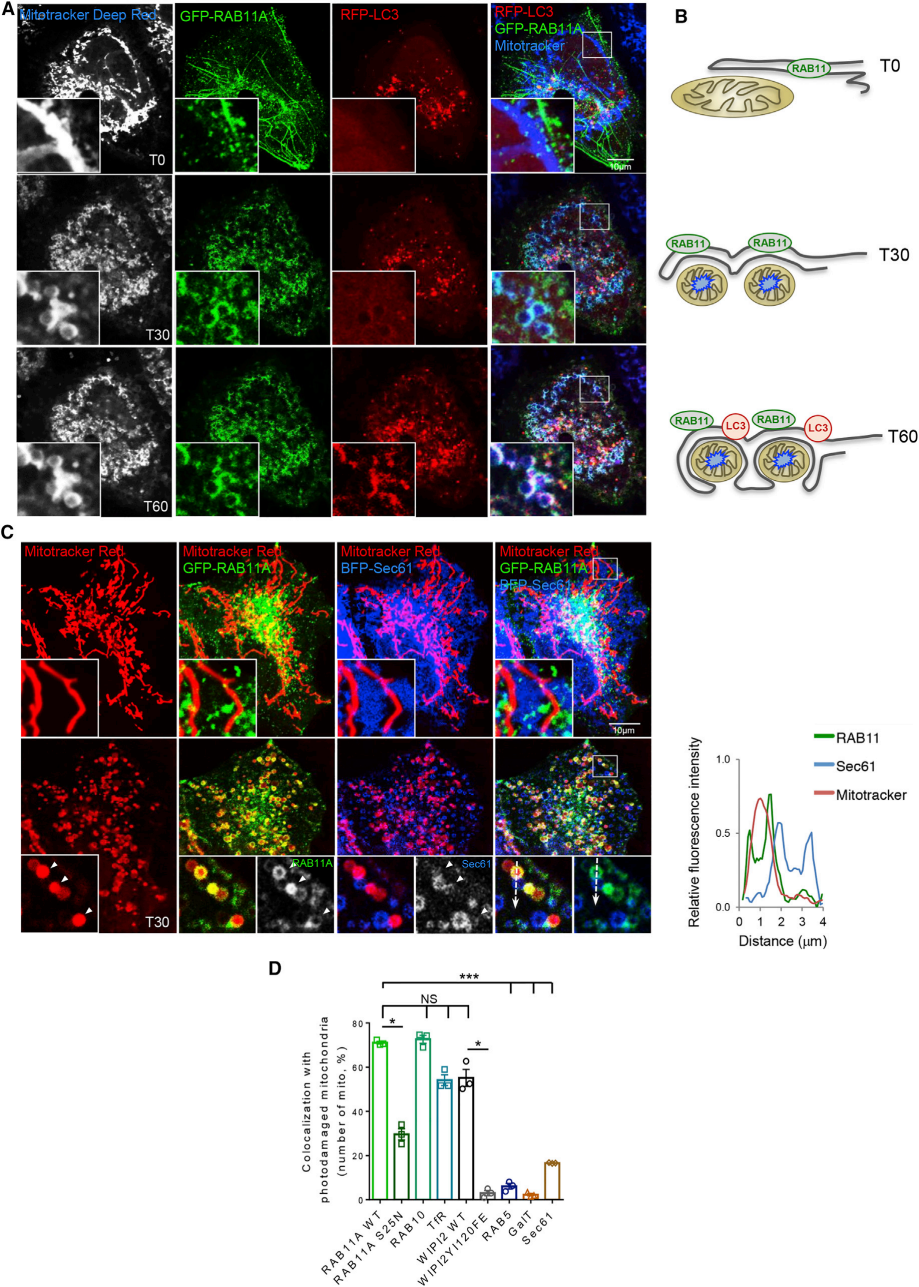
Photo-Damaged Mitochondria Are Engulfed by RAB11A-Positive Membranes (A and B) GFP-RAB11A HeLa cells transfected with RFP-LC3, incubated with MitoTracker Deep Red, and processed for live imaging. Mitochondria were photo-damaged using 488-nm laser light. See also Video S4. (C) GFP-RAB11A HeLa cells transfected with BFP-Sec61, labeled with MitoTracker Red, and processed as in (A). See also Videos S5 and S6. The relative fluorescence intensity of the area under the arrow is shown. Arrowheads indicate RAB11A engulfment of mitochondria with no ER. (D) The percentage of photo-damaged mitochondria surrounded by the different markers was measured in HeLa cells transfected as indicated and processed as in (A). Relevant images are shown in Figure S6. Data are means ± SEM, n = 3, 10 cells per condition; two-tailed paired t test; ^∗^p < 0.05; ^∗∗∗^p < 0.001; NS, not significant.

RAB11A-positive membranes associated with conventional cytoplasmic autophagic substrates, such as p62 (SQSTM1) and mutant huntingtin exon 1 (Figures S6A and S6B). In order to assess sequestration of a substrate by live imaging, we investigated mitochondrial autophagy (mitophagy), which shares its core machinery with canonical autophagy (Itakura et al., 2012). When mitochondria are damaged, autophagosomes form locally on their surface to form mitophagosomes (Youle and van der Bliek, 2012). Healthy mitochondria appear as tubular structures (Figures 6A and 6B). When mitochondria were exposed to 488-nm light that excites and damages their flavin-containing proteins, resulting in reactive oxygen species production (Kim and Lemasters, 2011), they became fragmented (Figures 6A and 6B, Videos S4 and S5). GFP-RAB11A decorated the surface of the damaged mitochondria 30 min after photo-damage, while RFP-LC3 started to decorate the photo-damaged organelle only at later time points (Figures 6A and 6B, Video S4). The RAB11A GTP binding-defective mutant RAB11AS25N (previously shown to be defective in WIPI2 recruitment/binding, Figures 1D and 1E) was not able to decorate the damaged mitochondria (Figures 6D and S6C). Interestingly, the photo-damage also caused fragmentation of the ER, but this remained distinct from the photo-damaged mitochondria (Figure 6C, Video S5). Both fragmented/damaged ER and mitochondria were surrounded by the RAB11A-positive membranes in discrete structures (Figure 6C and Video S5). We confirmed that RAB10, another recycling endosome marker, and WIPI2 appeared on photo-damaged mitochondria, while ER (Sec61), early endosome (RAB5), and TGN (GalT) markers did not (Figures 6D and S6D–S6G and Video S6). Consistent with a role for RAB11A in mitophagy, RAB11A silencing affected the recruitment of WIPI2 and recycling endosomes marker RAB10 on damaged mitochondria (Figures S6D and S6E) and the WIPI2 YI120FE RBD-defective mutant (Figures 6D and S6H) did not appear on photo-damaged mitochondria.

**Figure 7:**
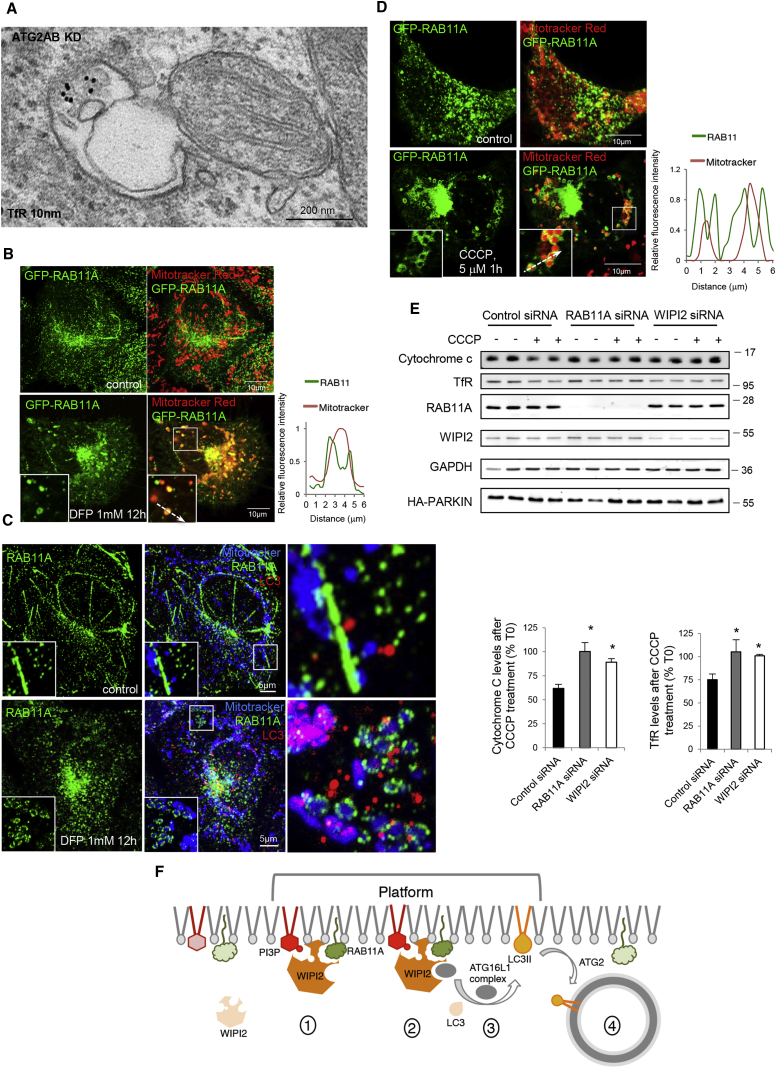
Mitochondria Are Engulfed by RAB11A-Positive Membranes during Parkin-Dependent and -Independent Mitophagy (A) HeLa cells treated with ATG2A/B siRNA were processed as in Figure 5D. (B) GFP-RAB11A HeLa cells treated with DFP, labeled with MitoTracker Red, and imaged. Line scan analysis of the area under the arrow is shown in the graph on the right. (C) HeLa cells treated as in (B) were stained for LC3 and RAB11A. Z-stacks of the area in the inset were processed by ZEN software and presented as max z-projected confocal images. (D) HA-PARKIN HEK cells transfected with GFP-RAB11A, labeled with MitoTracker Red, and treated with CCCP were imaged. Line scan analysis of the area under the arrow is shown in the graph on the right. (E) Lysates from HA-PARKIN HEK cells treated with control, RAB11A, or WIPI2 siRNA and incubated with DMSO or 5 μM CCCP for 6 hr were probed as indicated. Data are means ± SD, n = 3; two-tailed unpaired t test, ^∗^p < 0.05. (F) The cartoon summarizes events that lead to formation of the autophagosome: coincident detection of PI3P and RAB11A by WIPI2 (1) and recruitment of ATG16L1 complex (2–3). Here we show that all the relevant events preceding the completion of autophagosome formation (4) occur on the same membrane district (the platform), the RAB11A-positive membranes. Light green, inactive RAB11A, dark green, active RAB11A.

Membranes containing TfR also decorated damaged mitochondria (Figures 6D and S6I) and EM confirmed that double-membrane structures labeled by TfR (likely phagophores) engulf damaged mitochondria in cells where autophagosome closure was impaired by ATG2A/B silencing (Figure 7A).

In cells defective in autophagosome completion (by means of ATG5, ATG7, or ATG7/ATG10 silencing), the RAB11A compartment is able to decorate photo-damaged mitochondria normally (Figures S7A–S7C), while in cells defective in machinery required for earlier stages of autophagosome formation (ULK1 and VPS34 complex components) RAB11A-positive membranes failed to decorate damaged mitochondria (Figures S7D–S7F). Thus, recruitment of RAB11A-positive membranes to damaged mitochondria may be downstream of the ULK1 and VPS34 complexes but upstream of the ATG conjugation system (Figure S7).

When mitophagy was induced by CCCP treatment in HEK cells stably expressing HA-PARKIN (Narendra et al., 2008) or by treating cells with the iron chelator deferiprone (DFP) (Allen et al., 2013), the RAB11A compartment was also able to decorate the damaged mitochondria (Figures 7B–7D). Consistent with this, depletion of RAB11A and WIPI2 delayed degradation of depolarized mitochondria (Figure 7E). Interestingly, mitophagy induction caused TfR degradation, as this transmembrane protein follows the destiny of the recycling endosome membranes (Figure 7E). Thus, these data suggest that the RAB11A-positive membranes are the platform where the autophagosomes form to engulf substrates and damaged organelles.

## Discussion

Historically, the formation of PI3P at the IM sites and the recruitment of PI3P-binding autophagy proteins (WIPI2 and DFCP1) have been considered as key events associated with the initiation of autophagosome biogenesis (Nascimbeni et al., 2017). Here we describe the interaction of WIPI2 with the recycling endosome GTPase RAB11A as a critical step that enables the recruitment of the autophagy machinery, such as ATG16L1, to specify the sites of autophagosome formation on RAB11A-positive membranes. Indeed, as most phosphoinositide-protein interactions have weak binding affinities, dual recognition of phosphoinositides and membrane-associated proteins (GTPases) increases local binding strength and ensures more specific definition of the recruitment sites (Axe et al., 2008, Carlton and Cullen, 2005, Dooley et al., 2014, Vicinanza et al., 2015). As a mechanism of precise targeting, coincident detection is a recurring theme in membrane trafficking. Here, we show that the PI3P signal is necessary but not sufficient to stabilize WIPI2 binding at the isolation membrane/RAB11A-positive membranes (Figure 7F).

As PI3P is enriched in many compartments (i.e., early endosomes where some autophagy-core proteins [i.e., WIPI2 or ATG16L1] do not traffic; Figures S1F and S1G and Puri et al., 2013), it is likely that RAB11A acts in concert with a localized pool of PI3P to facilitate WIPI2 recruitment and to specify the membranes to which it binds (likely to be the “IM”). The interactions between RAB11A-WIPI2-ATG16L1 thus suggest a new model for autophagosome biogenesis where RAB11A-positive membranes provide a platform on which autophagosome assembly occurs (Figure 7F). Indeed, as the RAB11A-WIPI2 interaction is enhanced in cells where the autophagosome completion is impaired (ATG16L1-depleted cells), it is possible that the ability of WIPI2 to recycle off the RAB11A compartment is dependent on completion of later stages of autophagosome formation (e.g., LC3 membrane conjugation). Indeed, in the absence of ATG16L1, WIPI2-positive precursor structures accumulate on the RAB11A compartment (Figure 2G).

Traditionally, RAB11A-positive recycling endosomes are considered to mediate the slow recycling to the cell surface of certain endocytosed molecules, such as the transferrin receptor. Our data suggest that RAB11A-positive membranes serve as a platform for assembling autophagosomes. These membranes are likely to be recycling endosomes, as opposed to other RAB11A-positive compartments, as autophagic machinery also colocalizes with other recycling endosome markers, like RAB10 and transferrin and its receptor. Thus, we are proposing a novel function of this compartment. This model was corroborated by confocal, superresolution structure illuminated microscopy and electron microscopy (whole-mount EM, pre-embedding, immuno-gold labeling on cryosections), as well as a range of biochemical approaches, including isolation of the RAB11A compartment with Ferrofluid-Tf approaches (Li et al., 2005). Consistent with these data, we found that autophagic substrates were enwrapped and degraded by RAB11A-containing membranes. Importantly, the association of the RAB11A compartment with mitochondria, damaged by several stimuli, precedes LC3 association to these sites and is important for degradation of damaged mitochondria by mitophagy (Itakura et al., 2012, Kim and Lemasters, 2011, Youle and van der Bliek, 2012). Furthermore, the transferrin receptor, an integral membrane protein, which localizes predominantly to RAB11A-positive recycling endosomes during various autophagy induction conditions, is also degraded by autophagy, clarifying a new route for degradation of this receptor.

Our data do not contradict previous studies reporting phagophores being localized in proximity to other compartments, like the ER/mitochondria contact sites, but do enable the crucial distinction between membranes that contribute to autophagosomes, versus the platform on which autophagosome formation/LC3 lipidation occurs. Previous literature has repeatedly reported that ER-associated structures, including MAM, ERGIC (ER-Golgi intermediate compartment), and ER exit sites, are relevant to autophagosome formation, and that the IMs appear in very close proximity to the ER/MAM (Hamasaki et al., 2013, Lamb et al., 2013, Uemura et al., 2014). Furthermore, omegasomes have been described as tubular structures adjacent to and in contact with the ER (Uemura et al., 2014). This is exactly what we see – autophagosome formation occurs on tubular RAB11A-positive membranes adjacent to the ER/MAM. Recent data suggest, however, that the autophagosome formation sites (marked by ATG16L1 and related proteins) are largely distinct from the ER exit sites, the ERGIC compartments (Karanasios et al., 2016), and the MAM (Garofalo et al., 2016, Hamasaki et al., 2013), even while they are very close by. Indeed, the very close proximity between the ER and the recycling endosomes is compatible with previous observations. Importantly, our data do not contest other assertions that non-recycling endosome membranes contribute to autophagosomes, or that they may provide important functions, including LC3-lipidating activities. Interestingly, we observed that ATG3, which is critical for LC3 lipidation is more associated with the ER/MAM compartments than the RAB11A-positive membranes (Figures 3A and S3F), compatible with previous data suggesting that there is a migration of components of the lipidation machinery to the sites of autophagosome biogenesis (Ge et al., 2013, Ge et al., 2014). Since ATG3 is the E2-like enzyme that enables LC3 lipidation, it may be desirable for the cell to be able to regulate this process by trafficking ATG3 to the recycling endosomes, rather than having it resident there constitutively. Such processes are expected and will be required to enable controlled evolution of autophagosomes from RAB11A-positive compartment.

Importantly, ATG2, required for the completion of IM closure (Tamura et al., 2017, Velikkakath et al., 2012), was found enriched in the bound/RAB11A membrane fraction from the Ferrofluid assay (Figure 5H). Furthermore, ATG2 depletion led to autophagosome accumulation at their site of formation, the RAB11A-positive recycling endosomes (Figures 4G and 4H).

This supports the idea that key events preceding the completion of autophagosome formation occur on the same RAB11A-positive membrane subcompartment (the platform) (Figure 7F). Our data suggest that late stages of autophagosome formation occur on RAB11A-positive membranes and this compartment also recruits machinery required for earlier events in the process. Importantly, WIPI2 puncta form on these membranes prior to LC3 recruitment. While we are arguing that the RAB11A-positive membranes represent a platform for autophagosome formation because this is where the key events of LC3 association occur (which define autophagosomes), we believe that it is likely that the process requires membrane inputs from other compartments for delivery of key machineries and enzymes. But importantly, these inputs likely cooperate to form autophagosomes, which evolve primarily from RAB11A-positive membranes.

Our demonstration that RAB11A-positive membranes may be ideally suited to serve as a platform for the binding of ATG proteins during the assembly of autophagosomes calls for a reconsideration of the roles of this compartment and how its regulation may impact autophagy. We think that it is very likely that the autophagic machinery enables the maturation of RAB11A-positive membranes into autophagosomes, which will have different compositions to their platform membranes, and that the WIPI2-RAB11A interaction (along with PI3P) helps to dictate which subdomains of the recycling endosome are modified in this way. Thus, our findings raise new questions about how autophagosomes form, and what the key membrane sculpting events are.

## STAR★Methods

### Key Resources Table

**Table:** 

REAGENT or RESOURCE	SOURCE	IDENTIFIER
**Antibodies**		

rabbit anti-Alexa488	Invitrogen	Cat#(A11094) AB_221544
rabbit anti-ATG2A	Cell Signaling	Cat#( (15011S)
rabbit anti-ATG3	ABCAM	Cat# (ab108251) AB_10865145
rabbit anti-ATG5	SIGMA	Cat# (A0856) AB_1078238
rabbit anti-ATG7	ABCAM	Cat# (ab52472) AB_867756
rabbit anti-ATG10	ABCAM	Cat# (ab124711) AB_10974774
mouse anti-ATG14	MBL	Cat# (M184-3) AB_10897331
rabbit anti-Phospho-ATG14 (ser29)	Cell Signaling	Cat# (13155)
rabbit anti-ATG16L1	Cell Signaling	Cat# (D6D5) AB_10950320
rabbit anti-ATG16L1	MBL	Cat# (PM040) AB_1278757
rabbit anti-Beclin-1	ABCAM	Cat# (ab62472) AB_955697
mouse anti-calnexin	ABCAM	Cat# ab112995, AB_10860712
rabbit anti-calnexin	ABCAM	Cat# (ab133615)
rabbit anti-calreticulin	ABCAM	Cat# (ab4) AB_2069610
rabbit anti-Cherry	ABCAM	Cat# (ab183628) AB_2650480
mouse anti cytochrome C	BD	Cat# (556433) AB_396417
rabbit anti-DFCP1	ABCAM	Cat# (ab90029) AB_2043225
mouse anti-EEA1	ABCAM	Cat# (ab706521)
rabbit anti-FIP200	Proteintech Europe	Cat# (100691)
rabbit anti-GABARAP	GENERON	Cat# (AP1821A) AB_2278762
rabbit anti-GABARAPL1	ABCAM	Cat# (ab86467)
mouse anti-GAPDH	ABCAM	Cat# (ab8245) AB_2107448
rabbit anti-GFP	Invitrogen	Cat# (A6455) AB_221570)
mouse anti-GFP	Clontech	Cat# (6322675)
mouse anti-HA	Biolegend	Cat# (16B12) AB_10063630
mouse anti-LC3B	Nanotools	Cat# (0231-100/LC3-5F10)
rabbit anti-LC3	NOVUS	Cat# (NBP-24689)
rabbit anti-LC3B	ABCAM	Cat# (ab192890)
rabbit anti-Mannosidase II	ABCAM	Cat# (ab107544) AB_10862721
mouse anti-P62	BD Transduction	Cat# (610832) AB_398151
rabbit anti-P62	MBL	Cat# (PM045) AB_1279301
mouse anti-PI3P	Echelon	Cat# (Z-P003) AB_427221
rabbit anti-RAB5	Cell Signaling	Cat# (3547P) AB_2300649
rabbit anti-RAB10	Cell Signaling	Cat# (D36C4) AB_10828219
rabbit anti-RAB11FIP1	Cell Signaling	Cat# (D9D8P)
rabbit anti-RAB11A	ABCAM	Cat# (ab128913) AB_11140633
mouse anti-rabbit conformation specific	Cell Signaling	Cat# (3678S) AB_1549606
rabbit anti-RFP	MBL	Cat# (PM005) AB_591279
rabbit anti-SEC23	SIGMA	Cat# (S7696) AB_1079893
rabbit anti-SEC61A	ABCAM	Cat# (ab183046) AB_2620158
rabbit anti-TGN46	Seaman et al. J Cell Sci. 2009	Cat# N/A
mouse-anti-human Transferrin Receptor	Fabbri et al., 2005	Cat# N/A
mouse anti-Transferrin Receptor	Life Tecnologies	Cat# (136-800) AB_86623
mouse anti-tubulin	SIGMA	Cat# (T9026) AB_477593
rabbit anti-ULK1	Cell Signaling	Cat# (4776S)
mouse anti-WIPI2	ABCAM	Cat# (ab105459) AB_10860881
rabbit anti-WIPI2	Cell Signaling	Cat# (8567)

**Chemicals, Peptides, and Recombinant Proteins**		

Ferrofluid EMG508	Megatech ltd.	TSD-06-24-11-1
AP21967	Clontech	635057
Bafilomycin A1	Enzo	BML-CM110
DFP	Sigma	379409
CCCP	Sigma	C2759
SMER28	Tocris	4297
VPS34-IN1	Calbiochem	532628
Nocodazole	Sigma	M1404
HIS-RAB11A	Biorbyt	orb81295
GST-WIPI2	Abnova	H00026100-P01
WIPI2-flag WT and mutants	This paper	N/A
ATG16L1-Flag	Ravikumar et al., 2010 NCB	N/A

**Critical Commercial Assays**		

GFP-TRAP	Chromotek	Gtma-20
LDH assay colorimetric	ABCAM	ab102526

**Experimental Models: Cell Lines**		

HeLa GFP-RAB11	Puri et al., 2013 Cell	N/A
HeLa GFP-WIPI2	Vicinanza et al., 2015 Mol. Cell	N/A
HeLa CRISPR/Cas9 ATG16L1 knockout cell lines	Bento et al., 2016 Nature Comm.	N/A
HEK HA-PARKIN	Kruppa et al., 2018 Dev. Cell.	N/A
HeLa	ATCC	CCL-2
SHSY5Y	ATCC	CRL-2266
ATG16 null MEF	Fujita et al., 2009 JBC	N/A

**Experimental Models: Organisms/Strains**		

C57BL/6 mice	Jackson Laboratories	C57BL/6

**Oligonucleotides**		

Primer for WIPI2 LE115AT mutant CAGAGGCTGATAGTATGCGCACGGAGTCCCTGTACATCCAC	This paper	N/A
Primer for WIPI2 YI120FE mutant (CCTGGAGGAGTCCCTGTTCGAGCACAACATTCGGGACATG)	This paper	N/A
Primer for WIPI2R108E mutant (GCTGTGAAGCTCAACGACCAGAGGCTGATAGTATG)	This paper	N/A
Primer for RAB11S25N mutant (GGTGTTGGAAAGAATAATCTCCTGTCTCG)	This paper	N/A
Primer for RAB11Q70L mutant (TGGGACACAGCAGGGCTTGAGCGATATCGA)	This paper	N/A
For siRNA sequences see Table S1		

**Recombinant DNA**		

pEGFP-RAB11A	Puri et al., 2013 Cell	N/A
mCherry-RAB11A	In this paper	N/A
Flag-ATG4BC74A	Shu et al., 2010	N/A
pEGFP-WIPI2	T. Proikas-Cezanne (Eberhard Karls University Tübingen, Germany	N/A
FLAG-ATG16L1	Cadwell et al., 2008	N/A
pEGFP-LC3	Tamotsu Yoshimori (Osaka University, Japan)	N/A
pECFP-LC3	Tamotsu Yoshimori (Osaka University, Japan)	N/A
RFP-LC3	Tamotsu Yoshimori (Osaka University, Japan)	N/A
pEGFP-RAB5Q79L	Pietro De Camilli (Yale School of Medicine, USA)	N/A
GFP-FYVE4X	Pietro De Camilli (Yale School of Medicine, USA)	N/A
pEGFP-DFCP1	Nicholas Ktistakis (Babraham Institute, Cambridge, UK),	N/A
pEYFP-GalT	Jennifer A. Lippincott-Schwartz (NIH, Bethesda, USA)	N/A
pCMV-ATG7	Isei Tanida (Juntendo University School of Medicine, Japan)	N/A
BFP-SEC61	Addgene	49154
pEGFP-RAB10	Addgene	49472
RFP-RAB5A	Pietro De Camilli (Yale School of Medicine, USA)	N/A
PAM-Cherry-C1	Addgene	31929
PAM-Cherry-LC3	In this paper	N/A
pEGFP-FRB-RAB5	In this paper	N/A
RAB11FKBP-RFP	Prof.Dr. Casper Hoogenraad and Dr. Lukas C. Kapitein (Cell Biology, Utrecht University, The Netherland)	N/A
pFLAG-CMV-5a	Sigma	E7523
C1-mCherry	Clontech	632524

**Software and Algorithms**		

Prism 7	GraphPad	N/A
ZEN Black	Carl Zeiss Microscopy	N/A
Volocity	PerkinElmer	N/A
ImageJ	National Institute of Health, USA	N/A

### Contact for Reagent and Resource Sharing

Further information and requests for resources and reagents should be directed to and will be fulfilled by the Lead Contact, David C. Rubinsztein (dcr1000@cam.ac.uk).

### Experimental Model and Subject Details

#### Cell Culture

HeLa, SHSY5Y, MEF and HA-PARKIN HEK cells were cultured in Dulbecco’s modified Eagle’s medium (DMEM) (SIGMA D6548) supplemented with 2 mM L-glutamine, 100 U/ml Penicillin/Streptomycin and 10% Fetal Bovine Serum in 5% CO_2_ at 37°C. For primary cortical neurons, cortex was dissected from embryonic day 16.5 C57BL/6 mice cross and cultured in Neurobasal®-A Medium Minus Phenol Red (Life technologies), containing 1X B-27® Serum-Free Supplement (50X), Liquid (Life Technologies), 2mM Glutamine (SIGMA) and Penicillin-Streptomycin (SIGMA) (Pen 100U/ml-strep 0.1mg/ml). Cells are tested for mycoplasma contamination every two weeks. HeLa, SHSY5Y and HEK cells are female. The sexes of mouse-derived cells were not determined, as this was not considered to impact the basic cell biology we are describing.

#### Stable Cell Lines

HeLa cells were transfected with GFP-RAB11 construct (Puri et al., 2013). After 24 h, the cells were placed in selection medium (DMEM supplemented with 0.5 μg/ml G418) for 10 days. The antibiotic-resistant cells were then FACS sorted to isolate the GFP-expressing population. The cells were maintained in culture with DMEM supplemented with 0.5 μg/ml G418. HeLa cells stably expressing RFP-GFP tandem fluorescent-tagged LC3 or GFP-WIPI2 (Vicinanza et al., 2015) were maintained in culture with DMEM supplemented with 0.6 μg/ml G418, as previously described (Ravikumar et al., 2010). HeLa CRISPR/Cas9 ATG16L1 knockout cell lines were generated using a double-nicking strategy with paired guide RNAs to avoid off-target activity (Bento et al., 2016). HEK cells stably expressing HA-PARKIN were kindly provided by F. Buss and Antonina Kruppa (Kruppa et al., 2018).

### Method Details

#### Antibodies and Reagents

The antibodies listed in the Key Resources Table were used as follows: rabbit anti-Alexa488 (A11094-Invitrogen- 1:50 for EM), rabbit anti-ATG2A (15011S, Cell Signaling; ; 1:1000 for immunoblot and 1:200 for IF), rabbit anti-ATG3 (ab108251-ABCAM; 1:200 for IF), rabbit anti-ATG5 (A0856-SIGMA; 1:1000 for immunoblot), rabbit anti-ATG7 (ab52472–ABCAM; 1:1000 for immunoblot), rabbit anti-ATG10 (ab124711-ABCAM; 1:1000 for Blot), mouse anti-ATG14 (M184-3-MBL; 1:1000 for immunoblot and 1:200 for IF), rabbit anti-Phospho-ATG14 (ser29) (13155-Cell Signaling; 1:1000 for immunoblot), rabbit anti-ATG16L1 (D6D5-Cell Signaling f; 1:200 for immunofluorescence), rabbit anti-ATG16L1 (PM040-MBL ; 1:1000 for immunoblot) rabbit anti-Beclin-1 (ab62472-ABCAM; 1: 200 for IF), rabbit anti-calnexin (ab133615-ABCAM; 1:300 for IF), mouse anti-calnexin (ab112995-ABCAM; 1:400 for IF) rabbit anti-calreticulin (ab4-ABCAM; 1:1000 for immunoblot), rabbit anti-Cherry (ab183628-ABCAM; 1:50 for EM), mouse anti cytochrome C (556433-BD; 1:1000 for immunoblot), rabbit anti-DFCP1 (ab90029- ABCAM; 1:1000 for immunoblot), mouse anti-EEA1 (ab706521- ABCAM: 1:400 for IF and 1:1000 for immunoblot), rabbit anti-FIP200 (100691- Proteintech Europe; 1:1000 for immunoblot), rabbit anti-GABARAP (AP1821A-GENERON; 1:200 for IF), rabbit anti-GABARAPL1 (ab86467-ABCAM; 1: 200 for IF), mouse anti-GAPDH (ab8245-ABCAM; 1:5000 for immunoblot), rabbit anti-GFP (A6455-Invitrogen; 1:100 for EM), mouse anti-GFP (6322675-Clontech; 1:20.000 for immunoblot), mouse anti-HA (16B12- Biolegend; 1:400 for IF), mouse anti-LC3B (0231-100/LC3-5F10-Nanotools; 1:200 for IF), rabbit anti-LC3 (NBP-24689-NOVUS; 1:1000 for immunoblot and 1:200 for IF), rabbit anti-LC3B (ab192890-ABCAM; 1:200 for IF), rabbit anti-Mannosidase II (ab107544-ABCAM; 1:1000 for immunoblot), mouse anti-P62 (BD Transduction 610832; 1:200 for IF), rabbit anti-P62 (MBL PM045; 1:1000 for immunoblot) mouse anti-PI3P (Z-P003, Echelon; 1:300 for IF), rabbit anti-RAB5 (3547P- Cell Signaling; 1:1000 for immunoblot), rabbit anti-RAB10 (D36C4- Cell Signaling; 1:1000 for immunoblot), rabbit anti-RAB11FIP1 (D9D8P Cell Signaling; 1:1000 for immunoblot), rabbit anti-RAB11A (ab128913-ABCAM; ; 1:1000 for immunoblot and 1:200 for IF), mouse anti-rabbit conformation specific (3678S, Cell Signaling; 1:1000 for immunoblot), rabbit anti-RFP (PM005, MBL; 1:200 for IF), rabbit anti-SEC23 (S7696-SIGMA; 1:1000 for immunoblot), rabbit anti-SEC61A (ab183046-ABCAM; 1:1000 for immunoblot), rabbit anti-TGN46 (kind gift from Dr Matthew Seaman, University of Cambridge; 1:1000 for immunoblot), mouse-anti-human Transferrin Receptor (for immune-EM was a kindly gift from Dr Monica Fabbri (Fabbri et al., 2005)), mouse anti-Transferrin Receptor (136-800-Life Tecnologies ; 1:1000 for immunoblot and 1:200 for IF), mouse anti-tubulin (T9026-SIGMA; 1:4000 for immunoblot), rabbit anti-ULK1 (4776S-Cell Signaling; 1:200 for IF), mouse anti-WIPI2 (ab105459-ABCAM ; 1:200 for IF), rabbit anti-WIPI2 (8567-Cell Signaling ; 1:1000 for immunoblot), human transferrin Alexa-555 (T35352) and Alexa-488 (T13342) (Invitrogen). All Alexa-conjugated secondary antibodies and Mitotracker are from Invitrogen. Ferrofluid EMG508 is from Megatech ltd. (TSD-06-24-11-1). GFP-TRAP is from Chromotek. Protein-A gold is from CMC (Utrecht- NL).

Drug treatments used include: 250nM AP21967 (Clontech, 635057), 400 nM BAFA1 (Enzo, BML-CM110), 1mM DFP (Sigma, 379409), 5 μM CCCP (Sigma, C2759), 40 μM SMER28 (Tocris, 4297), 300nM VPS34-IN1 (Calbiochem, 532628) for 30 min. Microtubule depolymerization treatments include hypotonic medium (1:3 full medium:water) for 5 min or nocodazole (25 μM for 2 h).

#### Plasmids

pEGFP-RAB11A, mCherry-RAB11A, Flag-ATG4BC74A and pEGFP-WIPI2, have been described elsewhere (Cadwell et al., 2008, Puri et al., 2013, Shu et al., 2010, Vicinanza et al., 2015). pEGFP-WIPI2 was kindly provided by T. Proikas-Cezanne (Eberhard Karls University Tübingen, Germany), pEGFP-LC3, pECFP-LC3 and RFP-LC3 were a kind gift from Tamotsu Yoshimori (Osaka University, Japan), GFP-DFCP1 was a kind gift from Nicholas Ktistakis (Babraham Institute, Cambridge, UK), pEGFP-RAB5Q79L and GFP-FYVE4X were a kind gift from Pietro De Camilli (Yale School of Medicine, USA), pEYFP-GalT was a kind gift from Jennifer A. Lippincott-Schwartz (NIH, Bethesda, USA), pCMV-ATG7 was a kind gift from Isei Tanida (Juntendo University School of Medicine, Japan). BFP-SEC-61, pEGFP-RAB10 and RFP-RAB5A are from Addgene. The LC3 was sub-cloned from pEGFP-LC3 into PAM-Cherry-C1 using BglII EcoR1 restriction enzymes. To generate GFP-FRB-RAB5 was generated using NheI BglII from iRFP-FRB-RAB5 (Addgene 51612). The RAB11FKBP-RFP was kindly gift from Prof. Dr. Casper Hoogenraad and Dr. Lukas C. Kapitein (Cell Biology, Utrecht University, The Netherland). The pEGFP-WIPI2 mutants and pEGFP/mCherry RAB11 mutants were generated using QuikChange Multi Site-directed Mutagenesis Kit (Agilent Technologies, 200515-5). The primers used for WIPI2 LE115AT, YI120FE and R108E mutants or RAB11S25N or Q70L mutants are listed in the Key Resources Table. WIPI2 FRRG223FTTG mutant has been described elsewhere (Vicinanza et al., 2015). WIPI2 WT and mutants were sub-cloned into pFLAG-CMV-5a expression vector (Sigma) using EcoRI/BamHI restriction enzymes. RAB11 WT and mutants were sub-cloned into C1-mCherry expression vector (Clontech) using Xho/BamHI restriction enzymes.

#### Cell Transfection

The cells were seeded at 1-2 x10^5^ per 6-well and transfections were performed using LipofectAMINE 2000 for siRNA, according to the manufacturer’s instructions, using 100 nM siRNA. All the DNA constructs were transfected using Mirus Bio *Trans*IT ®-2020, at 1μg per well of a 6-well plate. The primary neurons were transfected with RFP-LC3 at the 9^th^ day of culture using LipofectAMINE 2000 following the manufacturer’s instructions.

#### Western Blot Analysis

HeLa cells were lysed in Laemmli buffer. Protein samples were boiled for 5–7 minutes at 100°C, separated by SDS-PAGE, transferred onto PVDF membranes, then subjected to western blot analysis and finally visualised using an ECL detection kit (GE Healthcare). Alternatively the membrane was labelled with fluorescent secondary antibody and analysed with a LICOR-Odyssey apparatus using IMAGE STUDIO Lite software, which enables quantitative analysis of blotting signals.

#### Immunoprecipitation

HeLa cells were treated as described in Figure legends and lysed in lysis buffer (50mM Hepes, 50mM NaCl, 10% glycerol, 1% Tryton X-100, 1.5mM MgCl, 5mM EGTA) for 15 min on ice and pelleted for 10min at 13.000 rpm. The supernatant was incubated with the WIPI2 antibody (8567 Cell Signaling) or control IgG antibodies (2729S Cell Signaling) (1:100) for 3 h and 2 h with Dynabeads Protein A (Novex-Lifechnologies). The immunoprecipitate was eluted by boiling the samples in Laemmli buffer for 5 min. GFP-tagged proteins (GFP-WIPI2, GFP-RAB11A) were pulled down using GFP-TRAP beads (ChromoTek) according to the manufacturer’s protocol. Proteins were resolved by SDS-PAGE.

#### Metabolic Labelling of Nascent Proteins by Click Chemistry

Metabolic labelling and click reactions were performed using the Click-iT Metabolic Labelling kit (Life Technologies, Grand Island, NY, USA) according to the manufacturer’s protocol. Cells were washed with warm PBS and cultured in 10% FBS DMEM (methionine-free) for 1 h to deplete the intracellular methionine reserves. Labelling of newly synthesized proteins was carried out in 10% FBS DMEM (methionine-free) containing 50μM AHA (L-azidohomoalanine, a methionine surrogate) for 2 hours. After labelling, the cells were lysed in RIPA buffer and AHA-labelled proteins conjugated to biotin. Equal amounts (250 μg) of proteins were tagged with the fluorescent dye and clicked proteins were precipitated. The samples were incubated at room temperature for 2 h with streptavidin magnetic beads and samples were analysed by SDS-PAGE.

#### Immunofluorescence Microscopy

Cells grown on coverslips at 25% confluency were fixed in 4% paraformaldehyde for 5 minutes, then permeabilised with 0.1% Triton. 1% BSA in PBS was used for blocking and for primary and secondary antibody incubations. The experiments visualizing endogenous LC3 were fixed with methanol for 5 minutes at -20°C. A Zeiss LSM710 or LSM880 confocal microscope was used for fluorescent confocal analysis. All confocal images were taken with a 63× oil-immersion lens.

#### Staining of PI3P for Immunofluorescence

HeLa cells were fixed in 2% paraformaldehyde and permeabilized with 20 μM digitonin in buffer A (20 mM Pipes pH 6.8, 137 mM NaCl, 2.7 mM KCl). Then cells were blocked with buffer A supplemented with 5% (v/v) FBS and 50 mM NH4Cl. Anti-PI3P antibodies from Echelon (for 1 h, 1:300) and secondary antibodies were applied in buffer A with 5% FBS. Cells underwent post-fixation for 5 min in 2% paraformaldehyde, were washed with PBS containing 50 mM NH4Cl, then were washed once with water and then mounted with Mowiol.

#### Artificial Localization of RAB11A to Early Endosomes (FRB-RAB5 FKBP-RAB11A)

HeLa were transfected for 20 h using Mirus Bio *Trans*IT ®-2020, at 0.7ug of FKBP-tagRFPt-RAB11 and 0.7ug of GFP-FRB-RAB5 per well of a 6-well plate. To couple FRB-RAB5 to FKBP-RAB11, a rapalog (AP21967, Clontech) was dissolved to 0.1 mM in ethanol and added in the culture medium for 30 min (250 nM). The cells were fixed and stained for anti-RFP (MBL) and endogenous WIPI2. As a control, a parallel set of cells were transfected with GFP-FRB-RAB5 and mCherry RAB11 (lacking the FKBP sequence) and treated with the rapalog AP21967 as in the previous experiment.

#### Live Cell Imaging

HeLa cells were seeded on MatTek Petri dishes (MatTek, Ashland MA USA) at a density of approximately 1.5 × 10^5^ cells per dish. Cells were placed in EBSS with HEPES, after which they were imaged immediately at 37°C. Imaging was performed on an incubated Zeiss AxioObserver Z1 microscope with a LSM780 confocal attachment using a 63× 1.4 NA Plan Apochromat oil-immersion lens. To image mitochondria, HeLa cells were loaded with Mitotracker Red CMXRos or Mitotracker Deep Red FM (Molecular probes) at 100 nM for 15 min in complete media. After two washes in fresh pre-warmed media, cells were shifted to EBSS–HEPES and imaged. To induce photo-damage of mitochondria, cells were exposed to 488-nm argon laser light at 100% power for 6 frames each lasting 30-60 seconds. After photo-irradiation, confocal images were collected every minute for up to 30 or 60 min. Alternatively, mitophagy was induced by 1mM Deferoxamine (dissolved in water) (DFP- SIGMA 379409) for 12 h or by 5μM CCCP (dissolved in DMSO) for 1 h.

#### Superresolution Microscopy

Samples were stained for conventional fluorescence microscopy and mounted on high-precision size 1.5 coverslips (Carl Zeiss Ltd, Cambridge). Coverslips were mounted with ProLong Gold anti-fade medium (Life Technologies, P36934) which was left to cure for 3 days at room temperature in order to produce samples with a consistent refractive index. Super-Resolution Structured Illumination Microscopy (SR-SIM) was performed using an Elyra PS1 instrument (Carl Zeiss Ltd), Samples were examined on the microscope using a 63x 1.4NA plan-apo Carl Zeiss objective lens and Immersol 518F (23°C) immersion oil. Image acquisition was carried out using ZEN 2012 Elyra edition software in which data sets were collected with 5 grating phases, 5 rotations and sufficient z positions spaced 110 nm apart to form an approximately 2 μm deep volume of raw SR-SIM data. Optimal grating frequencies were selected for each wavelength used. Structured Illumination post-processing was performed in ZEN using parameters determined by automated analysis of the datasets. Reconstructed images were then corrected for spherical and chromatic aberrations using channel alignment information, which was created using a 3D array of multi-spectral beads previously imaged with the same instrument settings. The average final image resolution was calculated to be 110 nm in x and y dimensions and 240 nm in the z dimension which represents a two-fold lateral and axial improvement in resolution compared to conventional microscopy.

#### Immunogold Electron Microscopy

##### Immunogold on Cryosections

HeLa cells were transfected with pEGFP-LC3 or Flag-ATG4B C74A for 24 h. The HeLa cells transfected with the different constructs were starved 1 h in HBSS, loaded with anti-TfR antibody, or transferrin-Alexa488 for 1 h and then fixed with a mixture of 2% paraformaldehyde and 1% acrolein in phosphate buffer (pH. 7.4) for 2 h, at room temperature. Cells were then prepared for ultrathin cryosectioning and immunogold-labelled, as previously described (Puri et al., 2013). Briefly, fixed cells were washed once in PBS/0.02 M glycine, after which cells were scraped in 12% gelatin in PBS and embedded in the same solution. The cell-gelatin was cut into 1 mm blocks, infiltrated with 15% PVP in 1.7 M sucrose at 4°C overnight, mounted on aluminium pins and frozen in liquid nitrogen. Ultrathin cryosections were picked up in a mixture of 50% sucrose and 50% methylcellulose and incubated with primary antibodies (rabbit anti-GFP, rabbit anti-Alexa488 rabbit anti-mouse to recognize TfR, rabbit anti-Flag) followed by protein A gold (Utrecht). Double labelling was performed as previously described (Puri et al., 2013).

##### Pre-embedding

HeLa cells were starved in HBSS for 1 h and placed on ice and incubated with mouse anti-Transferrin receptor antibody followed by rabbit anti-mouse antibody and protein A gold 10nm. The cells were then placed at 37°C in HBSS for 1 h and fixed in a mixture of 2% Paraformaldehyde and 2% Glutaraldehyde in 0.1 M cacodylate buffer (pH 7.4) for 1 h at room temperature. The cells were then postfixed in 1% Osmium Tetroxide in 0.1 M cacodylate buffer (pH 7.4) for 20 min and processed for standard Epon embedding. The sections were observed using a Philips CM100 or FEI Tecnai Spirit electron microscopes.

##### Whole Mount EM

Whole mount EM was performed as described (Fujita et al., 2009, Stoorvogel et al., 1996). Briefly, HeLa cells transfected with PAM-cherry LC3 were cultured on gold grids carrying carbon-coated Formvar films. Soluble cytosolic proteins were removed by permeabilizing the cells in PBS, 1 mM EGTA, 0.5 mM MgCl2, 0.5 mg/ml saponin at 0°C. The cells were then washed with PBS, 1 mM EGTA, 0.5 mM MgC12, and fixed for 1 h at 4°C with 1% paraformaldehyde in PBS. The grids were transferred to blocking buffer (PBS, 0.5 mg/ ml saponin, 20 mM glycine, 0.1% cold water fish gelatin (Sigma G-7765), 0.02% NaN2. The cells were immuno-double- labelled (Stoorvogel et al., 1996) using anti RAB11A (5-nm protein A gold) and anti GFP (10-nm protein A-colloidal gold). The cells were fixed with glutaraldehyde after each of the sequential labeling steps to permanently immobilize the colloidal gold label and to exclude cross-labelling (Stoorvogel et al., 1996). Control cells silenced for RAB11A for 5 days and not transfected with PAM-cherry LC3 were treated with the same immunolabelling procedure.

#### LC-MS/MS

Samples were resolved a short distance into a pre-cast minigel, the entire lane was excised and cut into 4 approximately equal sized chunks. The proteins were reduced, alkylated and digested in-gel with the resulting tryptic peptides analysed by LC-MSMS using an OrbiTrap XL (Thermo Scientific) coupled to a nanoAcquity UHPLC (Waters). Raw files were converted to mzML using MSConvert (Proteowizard) and searched against a human Uniprot database (downloaded 090614, 20,264 entries) using MASCOT 2.3. Deamidation (N,Q) and oxidation (M) were set as variable modifications and carbamidomethylation (C) as a fixed modification. Peptide and protein identifications were validated in Scaffold 4.3.2. Peptide identifications greater than 90% probability, as established by Peptide Prophet, were accepted. Protein identification required greater than 95% probability and a minimum of 2 peptides.

#### Multiple Sequence Alignment

The multiple sequence alignment was performed with Clustal Omega1 and figures prepared with ESPript 3.02. The figures are coloured according to the physicochemical properties of the residues and boxes highlight total or partial conservation between sequences.

#### Isolation of Recombinant WIPI2 Proteins

C-terminal tagged WIPI2 WT and WIPI2 mutants were purified from HeLa cells transfected with WIPI2 WT, WIPI2 LE115AT WIPI2 YI120FE and WIPI2 FRRG223FTTG FLAG-tagged plasmids. WIPI2 and ATG16L1 were purified from cell lysates with anti-FLAG-affinity beads (Sigma, #A2220) in FLAG lysis buffer (20 mM Tris-HCl, pH 6.8, 137 mM NaCl, 1 mM EGTA, 1% Triton x100, 1mM DTT, 10% Glycerol and protease inhibitors cocktail). After washing with the FLAG-lysis buffer, the proteins were eluted with 3X FLAG Peptides (#F4799, Sigma). Purified proteins were processed for SDS-PAGE and gels were stained with Coomassie Blue (Instant Blue, Expedeon) according to the manufacturer’s protocol.

#### WIPI2 Binding to Liposomes

The following phospholipids: brain L-α-phosphatidylcholine (L-α PC, Avanti # 840053), 1,2-Dioleoyl-sn-glycero-3-phosphoethanolamine (DOPE, Sigma #54008), 2-Oleoyl-1-palmitoyl-sn-glycero-3-phospho-L-serine (POPS, Sigma 51581), phosphatidylinositol 3-phosphate diC16 (PI3P diC16, Echelon, P-3016) and 1,2-dioleoyl-sn-glycero-3-[(N-(5-amino-1-carboxypentyl] iminodiacetic acid) succinyl (nickel salt) (18:1 DGS-NTA(Ni), Avanti # 790404) were dissolved in chloroform and mixed in molar ratios to generate different liposome populations:

##### RAB11A-Containing Liposomes

84%:10%:6% for L-αPC:DOPE:DGS-NTA(Ni) respectively (Figures 1B), 94%:4% for L-α PC: DGS-NTA(Ni) respectively (Figure 1H), 41.5%:52.5%:6% for L-αPC:PS: DGS-NTA(Ni) respectively (Figure 2I).

##### PI3P-Containing Liposomes

7.5%:30%:62.5% for PI3P:PS: L-α PC respectively (Figure 2H) or 56.5%:30%:7.5%/6% for L-αPC, PS, PI3P and DGS-NTA(Ni) respectively (Figure 2I);

##### RAB11A- and PI3P-Containing Liposomes

56.5%:30%:7.5%/6% for L-αPC: PS: PI3P : DGS-NTA(Ni) respectively (Figure 2I);

###### Empty Liposomes (Control PS Liposomes)

41.5%:52.5%:6% for L-αPC:PS: DGS-NTA(Ni) respectively (Figure 2I). Lipid films were dried under a stream of argon gas while they were rotated and were lyophilized overnight. Liposomes (multilamellar vesicles) were prepared from lipid films by vigorous vortexing in lipid hydration buffer (50 mM HEPES pH 7.4, 10 mM DTT and 5mM EGTA).

RAB11A-containing liposomes were generated by incubation 0.5 μM recombinant human HIS-RAB11A (Biorbyt #orb81295) with 500 μM liposomes for 20 minutes at room temperature in reaction buffer (50 mM HEPES pH 7.4, 1 μM DTT) to allow the immobilisation of the protein in liposomes through the reaction between the HIS tag and the NTA(Ni) group. The RAB11A-containing liposomes were collected by centrifugation (16,000g, 30 minutes) and resuspended in binding buffer (100mMKOAc, 2 mM Mg(OAc)_2_, 50 mM Hepes, 1mM DTT, 1mM MgCl_2_) at a final concentration of 500 μM.

Recombinant WIPI2 proteins were incubated with liposomes as indicated in binding buffer on ice for 5 minutes. WIPI2 binding was analysed by liposome sedimentation assay. GST-WIPI2 (Abnova #H00026100-P01), GST alone or WIPI2-flag proteins at approximate concentration of 150 nM were incubated with 30 μl liposomes (multilamellar vesicles, 500 μM). Then, the suspensions were centrifuged (16,000g, 30 minutes) and the liposome pellets were analysed by SDS-page to detect the membrane-bound fraction of WIPI2.

Binding of ATG16L1 (purified from HeLa cells expressing FLAG-tagged ATG16L1 using anti-FLAG M2 magnetic beads) to WT and mutants WIPI2 proteins was evaluated by incubation of recombinant proteins on ice for 5 min; this was followed by immunoprecipitation with anti-WIPI2 Rabbit antibodies (Cell Signaling, #8567) and SDS-PAGE analysis.

#### FerroFluid

Tf-enriched membrane isolation using Magnetic Microbeads (Ferrofluid) was performed as previous described (Li et al., 2005 Traffic). More details in Figure S5D.

#### LDH Activity

HeLa cells were treated as for Tf-Ferrofluid and the bound fraction was used to measure the LDH activity using LDH assay colorimetric Kit (ABCAM ab102526) following the manufacturer's instructions.

### Quantification and Statistical Analysis

#### Image Analysis

Volocity software (PerkinElmer) was used for analysis and processing of confocal images. For co-localization analysis of confocal images, we used Mander’s Coefficient. A minimum of 20 cells were examined each condition. All experiments were repeated at least three times. The background was fixed for all within-experiment analyses. Volocity software was also used for the Z stack reconstruction; the images presented also include the isosurface rendering in which there is no transparency and colocalised pixels do not appear as yellow.

For analysis of SIM images, final visualisation and video production was performed in Volocity 6.3 Software using isosurface rendering of selected cropped regions of the datasets. Isosurface rendering was used to more clearly visualise the shapes of fluorescent structures within the complex fluorescence volume datasets. This rendering type produces a three dimensional surface contour connecting points in space which have the same fluorescence intensity value (a threshold value), this value defines what is inside or outside of an object and allows us to visualise the point where the signal rises above background values. Structures rendered in this way appear solid and therefore what is inside or behind them is hidden from the viewer's perspective. It is not possible to directly observe colocalisation of signals in such images, instead we simply visualise the contact and overlap of the object's shapes.

To measure the percentage of photo-damaged mitochondria surrounded by the different markers, live cell images were scored for the presence or absence of markers on mitochondria after indicated treatments.

#### Statistics

Significance levels for comparisons between two groups were determined with t test (2 tail). ^∗^= p ≤ 0.05; ^∗∗^= p ≤0.01; ^∗∗∗^= p≤0.001. Significance levels for comparisons between more than two groups were determined with one-way ANOVA and multi-comparison analysis was performed with post hoc Tukey’s test ^∗^= p ≤ 0.05; ^∗∗^= p ≤0.01; ^∗∗∗^= p≤0.001; ^∗∗∗∗^= p≤0.0001. A P value of 0.05 was considered as the borderline for statistical significance.

For some experiments where we have assessed colocalisations in different conditions or western blot analysis, we have reported data from representative single experiments and noted cell numbers in the legends. This approach was used to minimise effects of heterogeneity between experiments.

Excel and Prism 7 software were used for statistical analysis and generation of graphs. See details in the figure legends.

## Supplemental Information

Document S1. Figures S1–S7

Table S1. siRNA Sequences, Related to STAR Methods

Video S1. Z-Stack Analysis of WIPI2 Localization on RAB11A-Positive Compartment in ATG16L1-Null Cells, Related to Figure 2 ATG16L1 CRISPR HeLa cells were transfected for 20 hr with GFP-WIPI2 (shown in red), fixed and stained for RAB11A (shown in green), and z-stack images collected by LSM880 confocal microscope. Z-stack images were processed by Volocity software (PerkinElmer) to assemble the movie.

Video S2. Superresolution Image of LC3 Vesicles on RAB11A-Positive Compartment, Related to Figure 4 HeLa cells were transfected for 20 hr with RFP-LC3 and GFP-RAB11A were fixed and visualized on an Elyra (Zeiss) superresolution microscope. Volocity software (PerkinElmer) was used to assemble the movie.

Video S3. WIPI2 Emerges from RAB11A-Positive Tubule and Matures into LC3-Positive Structure, Related to Figure 4 Confocal live imaging of HeLa cells treated with WIPI2 siRNA (siRNAb + siRNAc) and expressing GFP-WIPI2, the recycling endosome marker Cherry-RAB11A, and the autophagosome marker CFP-LC3. Cells were moved to EBSS-HEPES media and imaged for 30 min.

Video S4. Photo-Damaged Mitochondria Become Decorated by Ring Structures First Labeled by GFP-RAB11A and Then by RFP-LC3, Related to Figure 6 Confocal live imaging of HeLa cells expressing GFP-RAB11A and RFP-LC3 and loaded with MitoTracker Deep Red for 15 min. Cells were shifted to EBSS-HEPES media, photo-irradiated as described in STAR Methods and imaged for 60 min.

Video S5. Photo-Damaged Mitochondria Become Decorated by Ring Structures Positive for GFP-RAB11A and Negative for ER Marker Sec61, Related to Figure 6 Confocal live imaging of HeLa cells expressing GFP-RAB11A and BFP-Sec61 and loaded with MitoTracker Red for 15 min. Cells were shifted to EBSS-HEPES media, photo-irradiated as described in STAR Methods and imaged for 60 min.

Video S6. Photo-Damaged Mitochondria Become Decorated by Ring Structures Positive for GFP-RAB10 and Negative for ER Marker Sec61, Related to Figure 6 Confocal live imaging of HeLa cells expressing GFP-RAB10 and BFP-Sec61 and loaded with MitoTracker Red for 15 min. Cells were shifted to EBSS-HEPES media, photo-irradiated as described in STAR Methods and imaged for 60 min.

Document S2. Article plus Supplemental Information
