# Supplementary material for: The RAB11A-Positive Compartment Is a Primary Platform for Autophagosome Assembly Mediated by WIPI2 Recognition of PI3P-RAB11A
Source: Dev Cell. 2018 Apr 9;45(1):114–131.e8. doi: 10.1016/j.devcel.2018.03.008 (PMC5896254; doi:10.1016/j.devcel.2018.03.008)
Supplement: Document S1. Figures S1–S7 [file mmc1.pdf]

Developmental Cell, Volume *45*

Supplemental Information

The RAB11A-Positive Compartment Is a Primary Platform for Autophagosome Assembly Mediated by WIPI2 Recognition of PI3P-RAB11A

Claudia Puri, Mariella Vicinanza, Avraham Ashkenazi, Matthew J. Gratian, Qifeng Zhang, Carla F. Bento, Maurizio Renna, Fiona M. Menzies, and David C. Rubinsztein

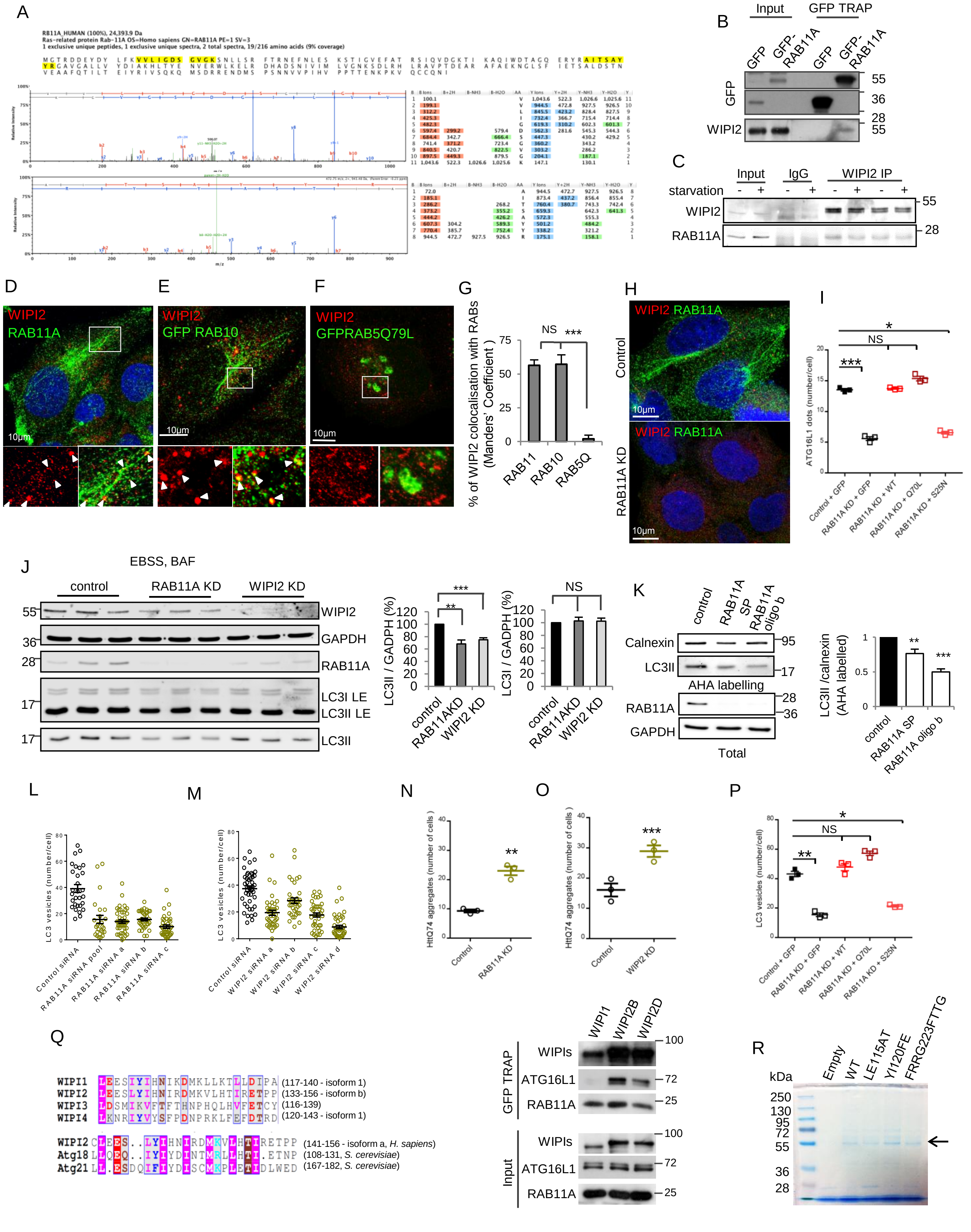

Puri, Vicinanza et al. Figure S1 (related to Figure 1) : WIPI2 and RAB11A interact and regulate autophagy

Figure S1 (related to Figure 1): WIPI2 and RAB11A interact and regulate autophagy. A) RAB11A was identified by LC-MSMS analysis in immunoprecipitates of GFP-WIPI2 from GFP-WIPI2 stable HeLa cells by 2 unique peptides, each with a peptide identification probability greater than 90% as determined by Scaffold. No RAB11A-derived peptides were identified in the control sample (GFP alone). Sequence coverage, spectra and fragmentation tables indicating the identified fragments are shown. **B**) GFP-RAB11A or GFP-empty vector was immunoprecipitated using GFP-TRAP from HeLa cells stably expressing GFP-RAB11A or GFP and blots were probed as indicated. **C**) HeLa cells were fed or starved for 2 h and endogenous WIPI2 was immunoprecipitated using specific antibodies. The immunoprecipitates were processed for immunoblotting and blots probed for endogenous WIPI2 or RAB11A. Normal IgG antibodies were used as control (IgG). Mouse anti-Rabbit conformation-specific secondary antibody was used to detect WIPI2 signal. **D**) HeLa cells were starved for 2h in EBSS and labelled for endogenous WIPI2 and RAB11A.**E-F**) HeLa cells were transiently transfected for 20 h with GFP-RAB10 (e) or GFP-RAB5Q79L (f), starved 1h and labeled for endogenous WIPI2.**G**) The histogram shows the quantification of the association of WIPI2 with RAB11A, GFP-RAB10 and GFP-RAB5Q79L (Manders' Coefficient). Data are mean ± SD (n=3 independent experiments, 20 cells counted each experiment and each condition). Two-tailed paired t test, ***=p<0.001, NS=not significant.**H**) HeLa cells treated with control or RAB11A siRNAs for 5 days were starved for 2 h in EBSS and labeled for endogenous WIPI2 and RAB11A.**I**) HeLa cells treated with RAB11A siRNAs for 5 days were transfected with GFP-empty or GFP-tagged RAB11A WT or mutants (RAB11AQ70L or S25N) for the last 24 h. Cells were starved for 2 h in EBSS, labeled for endogenous ATG16L1 and number of ATG16L1 structures quantified. Data are mean ± SEM (n=3 independent experiments, 50 cells analysed for each condition in each experiment); One-way ANOVA with post hoc Tukey's test, *=p<0.05, ***=p<0.001, NS=not significant. **J**) HeLa cells were transfected with control, RAB11A or WIPI2 siRNAs for 5 days, treated with EBSS containing 400 nM Bafilomycin A1 (BAF) for 4 h and processed for immunoblotting. The blots were probed for LC3, RAB11A, WIPI2 and GAPDH (loading control). The histogram shows the relative amount of LC3II (on the left) and LC3I (on the right) under BAF treatment normalized for GAPDH. Data are mean ± SD (One representative gel in triplicates is shown from 3 independent experiments); graphs represent means of the triplicates from three independent experiments; two-tailed paired *t*-test, **=p<0.01, ***=p<0.001, NS, not significant.**K**) HeLa cells treated with control or RAB11A siRNA (SmartPool or oligo b) were incubated with Click-iT® AHA (L-azidohomoalanine) for 2 h. Click chemistry was used to link the L-AHA azide group to a biotin labeled alkyne, rendering newly synthesized proteins labeled with biotin. These proteins were pulled down with streptavidin-conjugated magnetic beads and blotted for LC3. Calnexin was used as a control for L-AHA incorporation. Total lysates were processed and blotted as indicated to monitor the degree of RAB11A silencing. The histogram shows the relative amount of LC3II normalized for calnexin. Data are mean ± SD (n=3 independent experiments); two-tailed paired *t*-test, **=p<0.01, ***=p<0.001.**L-M**) HeLa cells transfected with control or deconvoluted RAB11A (**L**) or WIPI2 siRNA oligoes (**M**) for 5 days, were treated with BAF for 4 h, processed for immunofluorescence and labeled with endogenous LC3. The number of LC3 dots per single cell in each condition is shown. Data are mean ± SEM. (One representative experiment is shown, 30 cells counted for RAB11A siRNA, 40 cells counted for WIPI2 siRNA). **N-O**) HeLa cells were transfected with control or RAB11A siRNA (**n**) or WIPI2 siRNA (**o**) for 5 days and transiently transfected with HA-tagged Htt-Q74 (mutant form of huntingtin associated with Huntington's disease) for the last 48 h and processed for immunofluorescence. Percentage of cells with Htt-Q74 aggregates is shown in the graph. Data are mean ± SEM (n=3 independent experiments, 700 cells were analyzed for each condition in each experiment); two-tailed paired *t*-test; **=p<0.01, ***=p<0.001. **P**) HeLa cells treated with

RAB11A siRNAs for 5 days were transfected with GFP-empty or GFP-tagged RAB11A WT or mutants (RAB11AQ70L or S25N) for the last 24 h. Cells were starved for 4 h in EBSS, labeled for endogenous LC3 and number of LC3 structures quantified. Data are mean ± SEM (n=3 independent experiments, 50 cells analyzed for each condition in each experiment); One-way ANOVA with post hoc Tukey's test, *=$p<0.05$, **=$p<0.01$, NS=not significant. **Q**) Full sequence alignment shows strict identities and similarities between residues within WIPI family proteins (human and yeast orthologues) and residues within the RAB11-binding domain (only relevant regions are shown). GFP-TRAP of GFP-WIPIs was performed as in Figure 1A.**R**) Coomassie blue-staining of recombinant flag-tagged WIPI2 proteins (WT and mutants) after purification from HeLa cells using the anti-FLAG M2 Affinity gel and separation by SDS-PAGE.

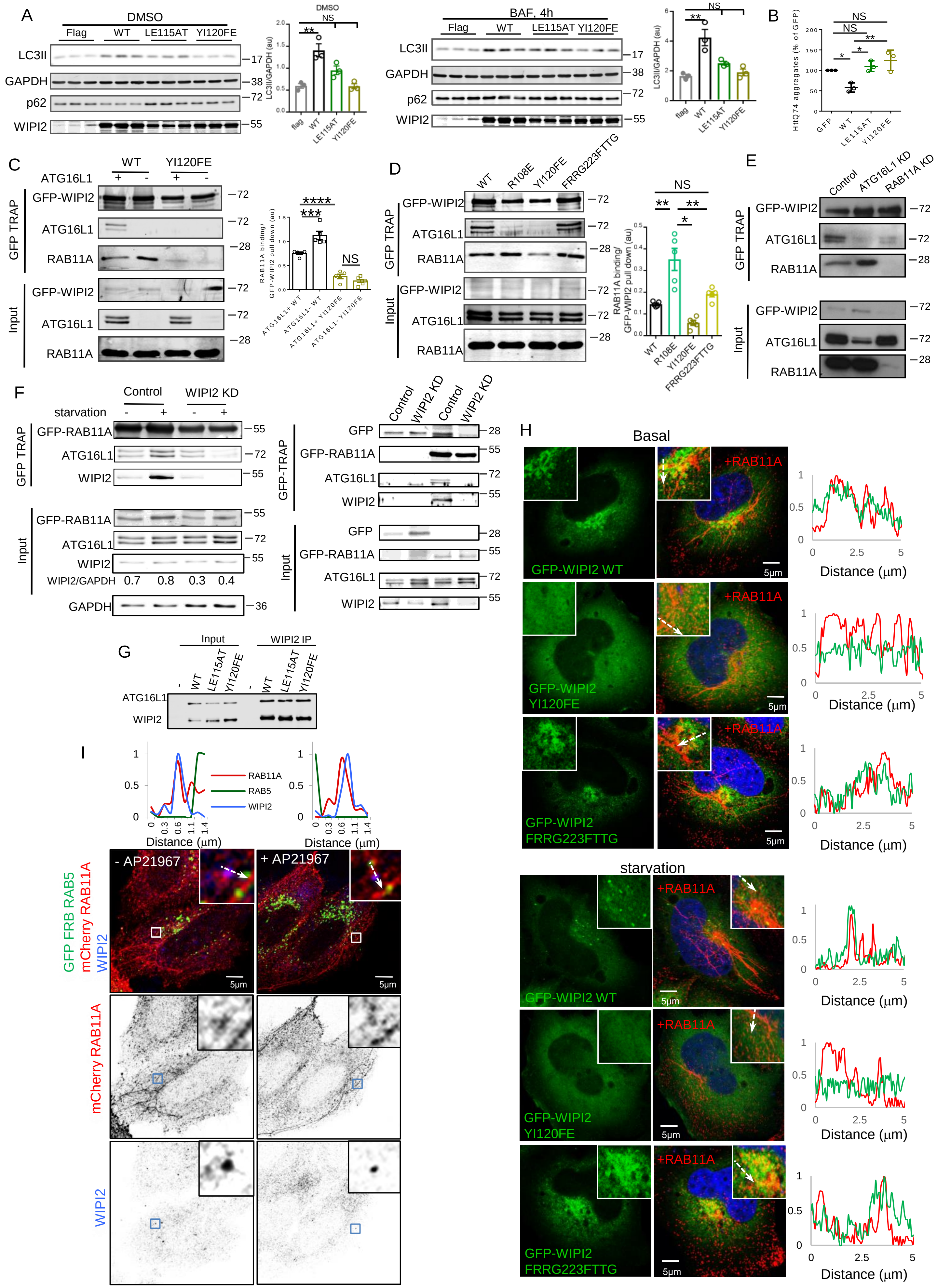

Puri, Vicinanza Figure S2 (related to Figure 2): Disruption of WIPI2-ATG16L1 interaction stabilizes WIPI2-RAB11A complex

Figure S2 (related to Figure 2): Disruption of WIPI2-ATG16L1 interaction stabilizes WIPI2-RAB11 complex.A-B) HeLa cells were transfected with GFP-empty or GFP-WIPI2 WT, LE115AT or YI120FE alone (**A**) or in combination with HA-tagged Htt-Q74 (mutant form of huntingtin associated with Huntington's disease) for the last 24 h (**B**). Cells were processed for western blotting to measure LC3II levels (**A**) or for immunofluorescence (**B**) to measure the percentages of transfected cells with aggregates. Data are mean ± SEM (n=3 independent experiments, 800 cells were analyzed for each condition in each experiment in (**B**)); One-way ANOVA with post hoc Tukey's test; *=p<0.05, **=p<0.01, NS=not significant.**C**) CRISPR/Cas9 ATG16L1 knockout and control cells (Bento et al., 2016) were transfected with GFP-WIPI2 WT or GFP-WIPI2 YI120FE for 20h. Immunoprecipitates obtained by GFP-TRAP were processed for immunoblot for RAB11A and ATG16L1. The amount of RAB11A co-immunoprecipitated with WIPI2 WT or YI120FE mutant in the presence or absence of ATG16L1 was quantified and normalized for the amount of WIPI2 immunoprecipitated. Data are mean ± SEM (n=5 independent experiments); One-way ANOVA with post hoc Tukey's test, ***=p<0.001, ****= p< 0.0001, NS=not significant.**D**) HeLa cells were transiently transfected with GFP-WIPI2 WT, R108E, YI120FE and FRRG223FTTG for 20 h. GFP-TRAP beads were used to isolate transfected proteins and protein complexes were analysed for ATG16L1 and RAB11A content. Quantification of RAB11A binding with WIPI2 WT or mutants is shown. Data are mean ± SEM (n=5 independent experiments); One-way ANOVA with post hoc Tukey's test, *=p<0.05, NS=not significant.**E**) HeLa cells were transfected with control, ATG16L1 siRNA or RAB11A siRNA for 5 days. In the last 20 h, the cells were transiently transfected with GFP-WIPI2 WT. GFP-TRAP was used on lysates as in b. **F**) HeLa cells treated with control or WIPI2 siRNA (oligo b and oligo c) were transfected in the last 20 h with GFP-RAB11A (left) or GFP-empty (right) and processed for GFP-TRAP immuno-precipitation. Blots were probed as indicated. Loading control for input was provided to show relative abundance of WIPI2 in control or WIPI2 siRNA-treated cells. Note that GFP-alone (in right hand panel lanes 1 and 2) does not pull-down ATG16L1 – the ATG16L1 runs as a doublet (due to different isoforms) in the GFP-RAB11A lane 3, but there is only a smaller single non-specific band in the GFP only pull-downs (lane 2). **G**) WIPI2 WT and mutants binding to ATG16L1 was evaluated in vitro using recombinant proteins.**H**) GFP-WIPI2 WT, YI120FE or FRRG223FTTG mutants were transiently transfected in HeLa cells. Cells were kept in complete media (Basal) or shifted to starvation media (2h, EBSS). Cells were fixed and labelled for endogenous RAB11A. Line scan analysis of selected areas (insets) for each condition is shown in the graph on the right (red line=RAB11A; green line=GFP-WIPI2 WT or mutants).**I**) HeLa cells transiently transfected with GFP-FRB-RAB5A and mCherry-RAB11A were treated with 250nM AP21967 for 30min in full-media and then fixed and stained for endogenous WIPI2. The graphs show the relative fluorescence intensity of the area under the arrows. Arrowheads show WIPI2 puncta on RAB11A and not on early endosomes by addition of AP21967.

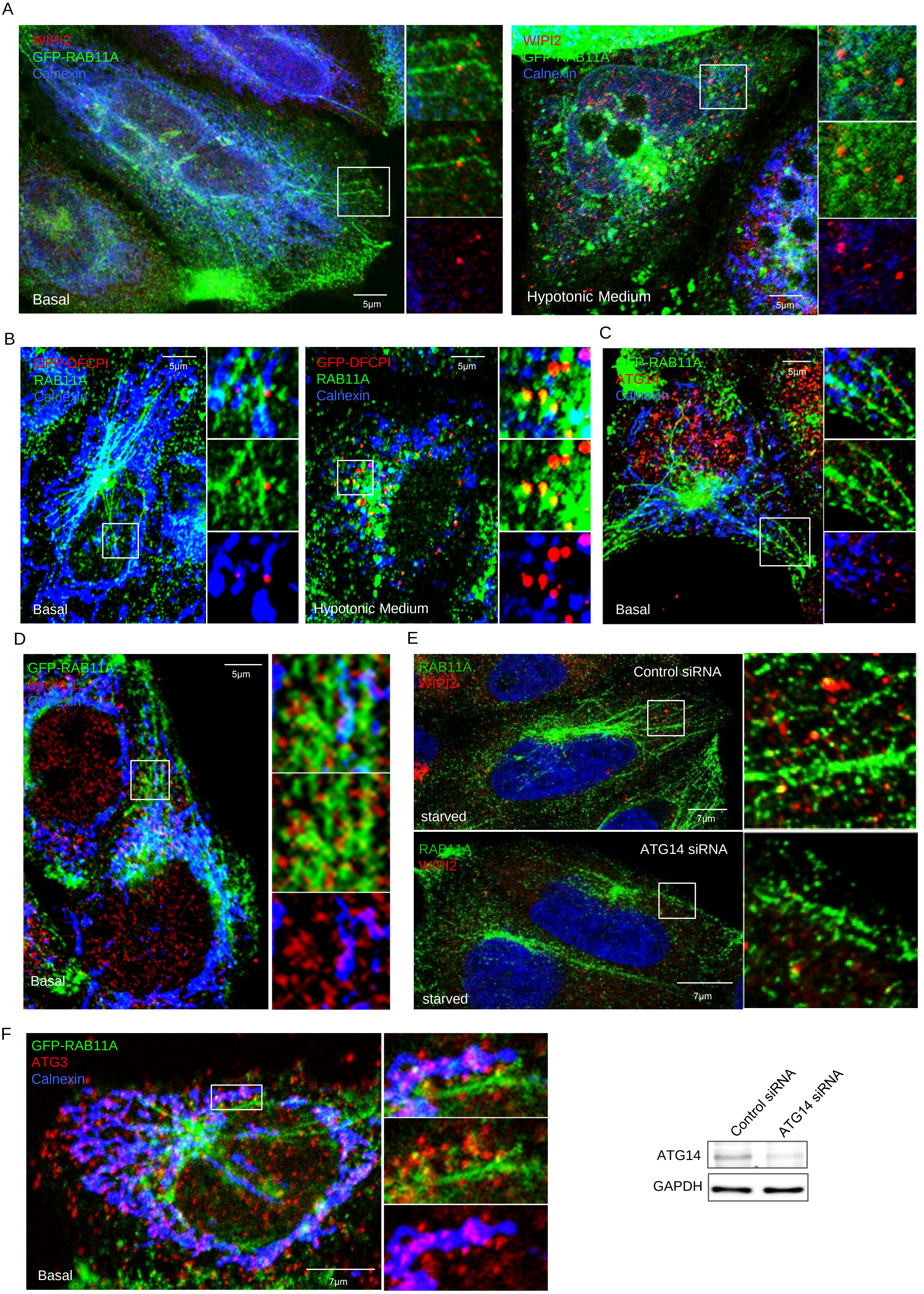

Puri , Vicinanza et al. Figure S3 (related to Figure 3): Autophagic proteins are on RAB11A compartments

Figure S3 (related to Figure 3): Autophagic proteins are on RAB11A compartments A) HeLa cells stably transfected with GFP-WIPI2 were fixed in basal conditions or under hypotonic medium treatment and labelled for endogenous RAB11A (red) and calnexin (blue). The quantification of the colocalisation is shown in the histogram in Figure **3A**. **B)** HeLa cells transiently transfected with GFP-DFCP1 were fixed in basal conditions or under hypotonic medium treatment and labelled for endogenous RAB11A (red) and calnexin (blue). The quantification of the colocalisation is shown in the histogram in Figure **3A**. **C)** HeLa cells stably transfected with GFP-RAB11A were fixed in basal conditions and labelled for endogenous ATG14 (red) and calnexin (blue). The quantification of the colocalisation is shown in the histogram in Figure **3A**.**D)** HeLa cells stably transfected with GFP-RAB11A were fixed in basal conditions and labelled for endogenous Beclin1 (red) and calnexin (blue). The quantification of the colocalisation is shown in the histogram in Figure **3A**.**E)** HeLa cells were transfected with control or ATG14 siRNA for 5 days and labelled for endogenous RAB11A and WIPI2 in starvation condition (EBSS, 2h). HeLa cells transfected with control or ATG14 were processed for immunoblotting to measure the degree of silencing.**F)** HeLa cells stably transfected with GFP-RAB11A were fixed in basal conditions and labelled for endogenous ATG3 (red) and calnexin (blue). The quantification of the colocalisation is shown in the histogram in Figure **3A**.

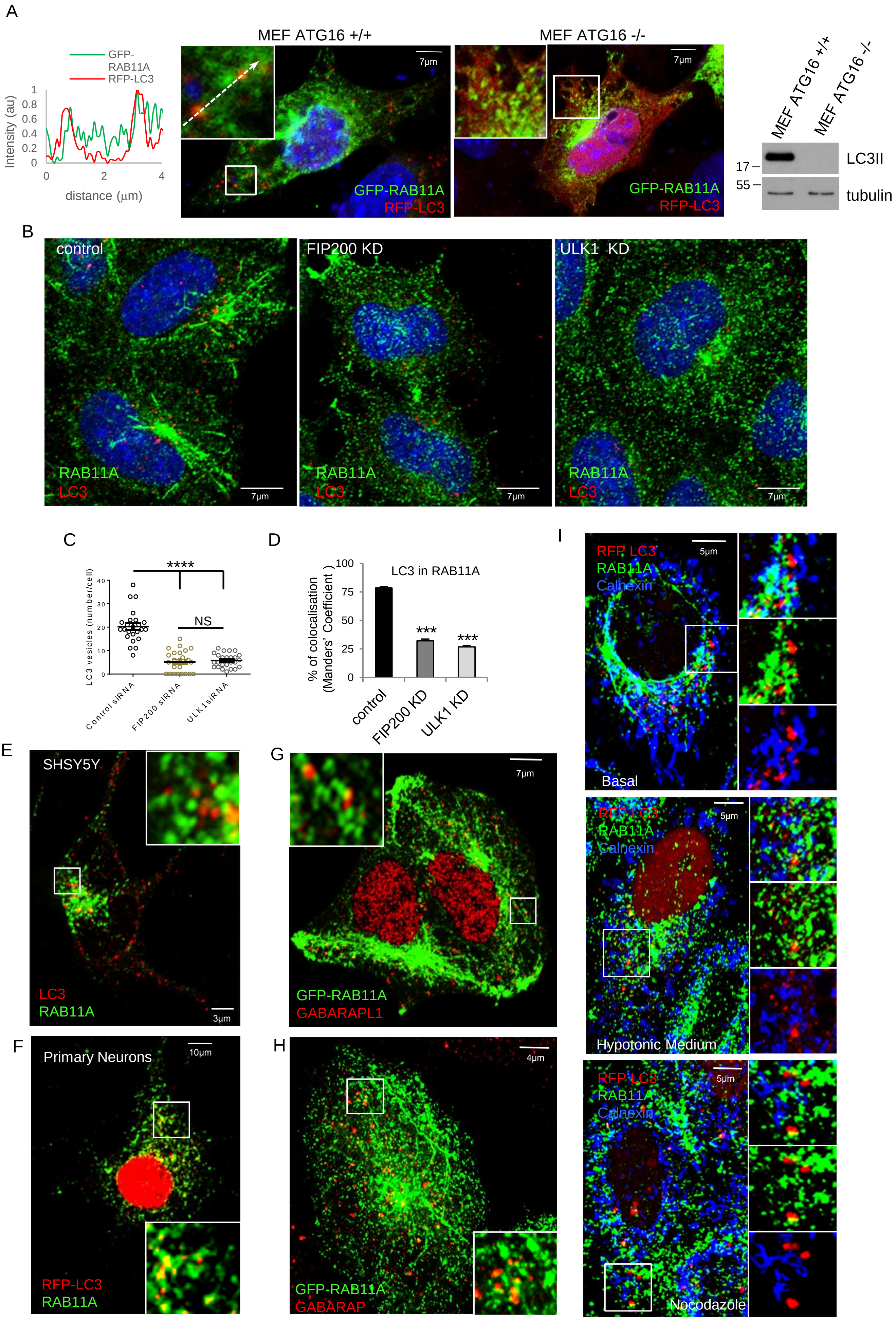

Puri, Vicinanza et al. Figure S4 (related to Figure 4) : Specificity of the LC3 vesicles on RAB11A-positive compartment

Figure S4 (related to Figure 4): Specificity of LC3 vesicles on RAB11A-positive compartment. A) Mouse embryonic fibroblasts (MEFs) from ATG16 control (+/+) and ATG16 null mice (-/-) were transfected for 20 h with mRFP-LC3 and GFP-RAB11A. The inserts show enlargements of the area in the white box, arrows indicate LC3 positive vesicles which are absent in the ATG16-/- MEFs, in which the RFP-LC3 demonstrates diffuse fluorescence. Line scan analysis of selected areas is shown in the graph. LC3 levels in both MEFs from ATG16 control (+/+) and ATG16 null mice (-/-) were measured by western blotting. **B-D)** HeLa cells were transfected with control or FIP200 or ULK1 siRNAs for 5 days and labelled for immunofluorescence. The histogram in (**c**) shows the LC3 dots in control and FIP200- and ULK1-depleted cells. Data are mean ± SEM (One representative experiment from 3 independent experiments, 28 cells were analysed). One-way ANOVA with post hoc Tukey's test, ****=p<0.0001, NS=not significant. The histogram in (**D**) shows the colocalisation of LC3 with RAB11A (Manders' Coefficient). Data are mean ± SEM (One representative experiment from 3 independent experiments, 28 cells were analysed); two-tailed paired *t*-test, ***=p<0.001.**E)** SHSY5Y cells were labeled for endogenous RAB11A and LC3.**F)** Mouse primary neurons were transfected with RFP-LC3 and labeled for endogenous RAB11A. Enlargements of the area in the white box are shown.**G)** HeLa cells stably expressing GFP-RAB11A were fixed and labeled for endogenous GABARAPL1. As seen for LC3, the GABARAPL1 positive vesicles decorate the RAB11A tubules.**H)** HeLa cells stably expressing GFP-RAB11A were fixed and labeled for endogenous GABARAP. As seen for LC3 and GABARAPL1, GABARAP-positive vesicles decorate the RAB11A tubules.**I)** HeLa cells were transfected for 20 h with RFP-LC3, treated or not with hypotonic medium (1:3 full medium:water) for 5 min or with nocodazole (25 µM for 2 h) and labelled for calnexin (MAM marker) (blue) and RAB11A (green). Relative quantification is presented in Fig. 4C.

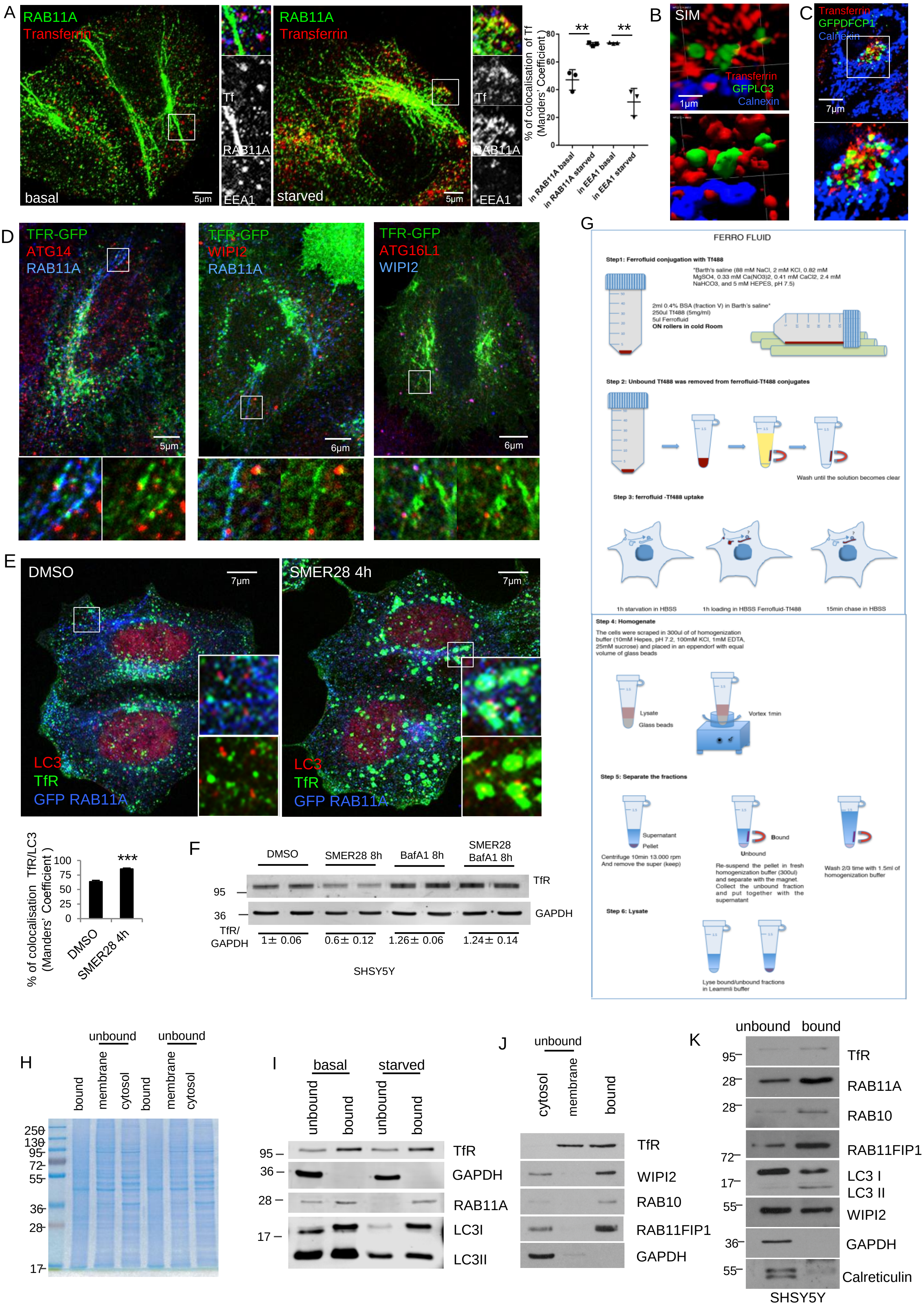

Puri, Vicinanza et al. Figure S5 (related to Figure 5): TfR is recruited to RAB11A/LC3 compartments at the onset of autophagy

Figure S5 (related to Figure 5) : TfR is recruited to RAB11A/LC3 compartments at the onset of autophagyA) HeLa cells were starved or not in HBSS for 1 h, loaded with transferrin Alexa555 for 1 h in HBSS or amino acid-rich, serum-free medium and chased for 15 min. The cells were then fixed and labelled with RAB11A and EEA1 (as early endosome marker). The histogram shows the colocalisation (Manders' Coefficient) of transferrin with RAB11A or EEA1. Data are mean ± SEM (Representative of three independent experiments, 23 cells analysed in basal and 24 cells analysed for starvation); two-tailed paired *t*-test, ***=p<0.001 .**B**) HeLa transfected for 20 h with GFP-LC3, starved in HBSS for 1 h, loaded with transferrin Alexa555 for 1 h in HBSS, chased for 15 min. and stained for calnexin were analysed for structural illumination (SIM) on Elyra (Zeiss) superresolution microscope.**C**) HeLa were transiently transfected for 20 h with GFP-DFCP1, loaded with TF-Alexa555 and stained for calnexin and analysed confocal microscope.**D**) HeLa cells transiently transfected for 20 h with TfR-GFP and starved for 1h were labelled for endogenous RAB11A and ATG14 or WIPI2 or ATG16L1.**E)** HeLa cells stably expressing GFP-RAB11A (blue) were treated 4 h with SMER28 or DMSO. The cells were loaded for the last 45 min with TfR antibody fixed and labeled for endogenous LC3. The histogram shows the colocalisation of TfR (green) in LC3 (red) in DMSO or SMER28-treated cells (Manders' Coefficient). Data are mean ± SEM (Representative of three independent experiments, 21 cells analysed in basal and 22 cells analysed for starvation); two-tailed paired *t*-test, ***=p<0.001.**F)** SHSY5Y cells were treated with DMSO or SMER28 or Bafilomycin A1 (BafA1) or SMER28 and BafA1 for 8 h. Control and treated cells were processed for immunoblotting. The number below the blot shows the TfR levels in treated cells compared with control (DMSO=1). Data are mean ± SD (representative of three independent experiments); two-tailed paired *t*-test, ***=p<0.001.**G)** The cartoon shows the different steps for the Ferrofluid-Tf Alexa 488 procedure used to isolate LC3-containing recycling endosomes. **H**) HeLa cells were processed as described in **G**. Bound and unbound fractions (cytosol and unbound membrane) were processed for SDS-PAGE and gels were stained with Coomassie Blue. Samples were loaded in duplicate.**I**) HeLa cells were starved or not in HBSS for 1 h, and processed as described in **G.J**) HeLa cells were processed as described in **G.** The unbound (cytosol and membrane) and bound fractions were collected and probed WIPI2 and other markers. Note WIPI2 in the unbound fraction localises in the cytoplasmic pool.**K)** SHSY5Y cells were starved 1 h in HBSS and loaded with Ferrofluid-Tf488 for 1 h in HBSS and chased for 15 min in HBSS. The cells were then fragmented and the membrane containing Ferrofluid-Tf Alexa 488 (bound) or not containing Ferrofluid-Tf Alexa 488 (unbound) were separated, lysed and analysed by immunoblotting.

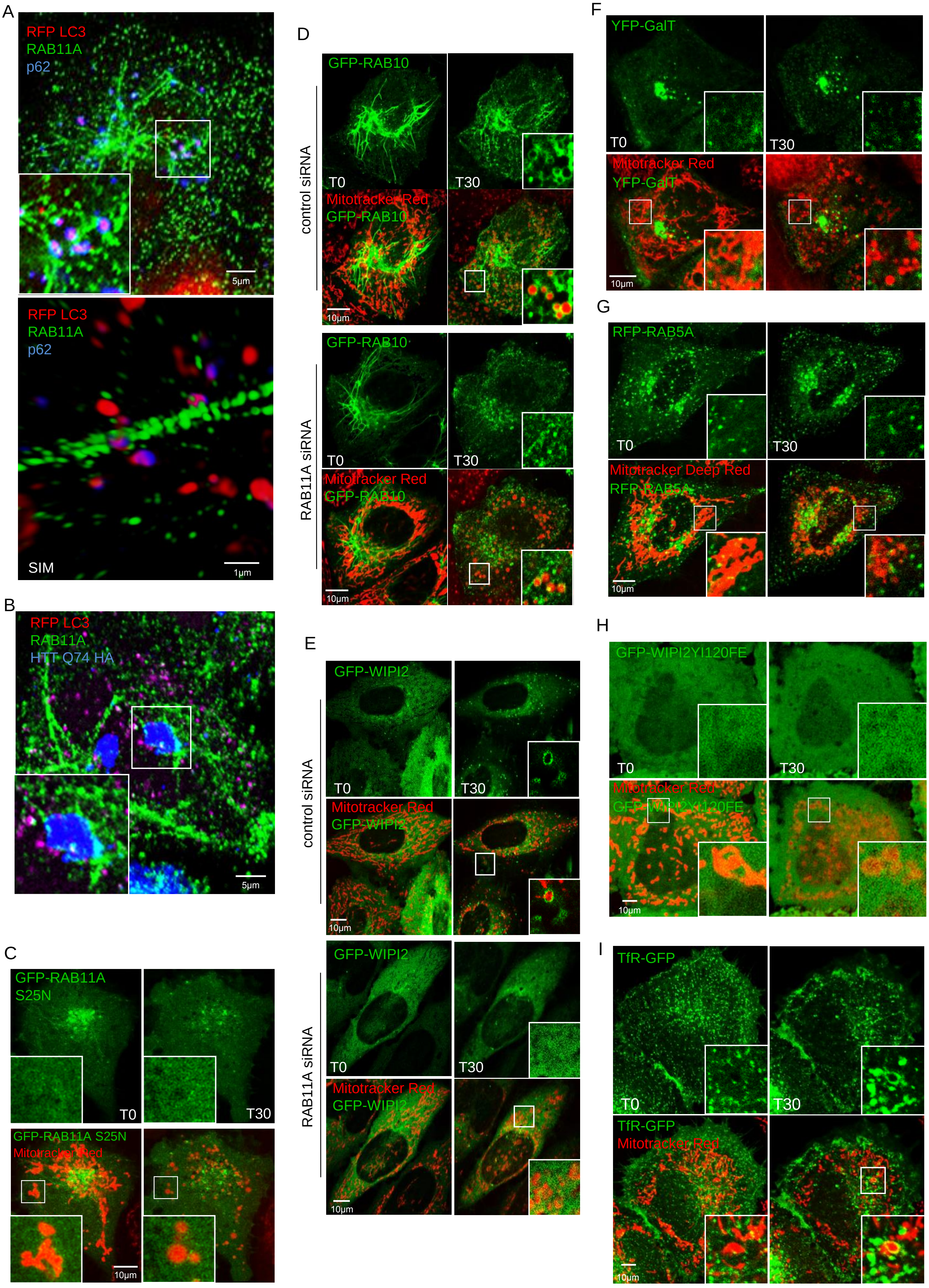

Puri , Vicinanza et al Figure S6 (related to Figure 6): Autophagic substrates are engulfed by RAB11A-positive compartment

Figure S6 (related to Figure 6): Autophagic substrates are engulfed by RAB11A-positive compartmentA) HeLa cells were transiently transfected for 20 h with RFP-LC3 and labelled for endogenous RAB11A and p62. Inserts show enlargements of the area in the white box. Bottom picture shows a similar experiment observed on Elyra (Zeiss) superresolution microscope (the structured illumination image).**B)** HeLa cells were transiently transfected for 48 h with Htt-Q74 HA-tagged and RFP-LC3 and labelled for endogenous RAB11A.**C**) HeLa cells transfected with GFP-RAB11A S25N were labelled with Mitotracker-Red, and processed for live imaging. Mitochondria were photodamaged using 488-nm light and recorded at T0 and after 30 min (T30).**D-E**) HeLa cells were transfected with control or RAB11A siRNA and transiently transfected with GFP-RAB10 (**D)** or GFP-WIPI2 (**E**) for 20 h. Cells were loaded with Mitotracker-Red and imaged as in (**C**). See also Movie 6.**F-I**) HeLa cells transfected with YFP-GalT (**F**), RFP-RAB5A (**G**), GFP-WIPI2 YI120FE (**H**) or TfR-GFP (**I**) were labelled with Mitotracker-Deep Red (**G**) or Mitotracker-Red (**F**) and (**H**) and imaged as in (**C**).

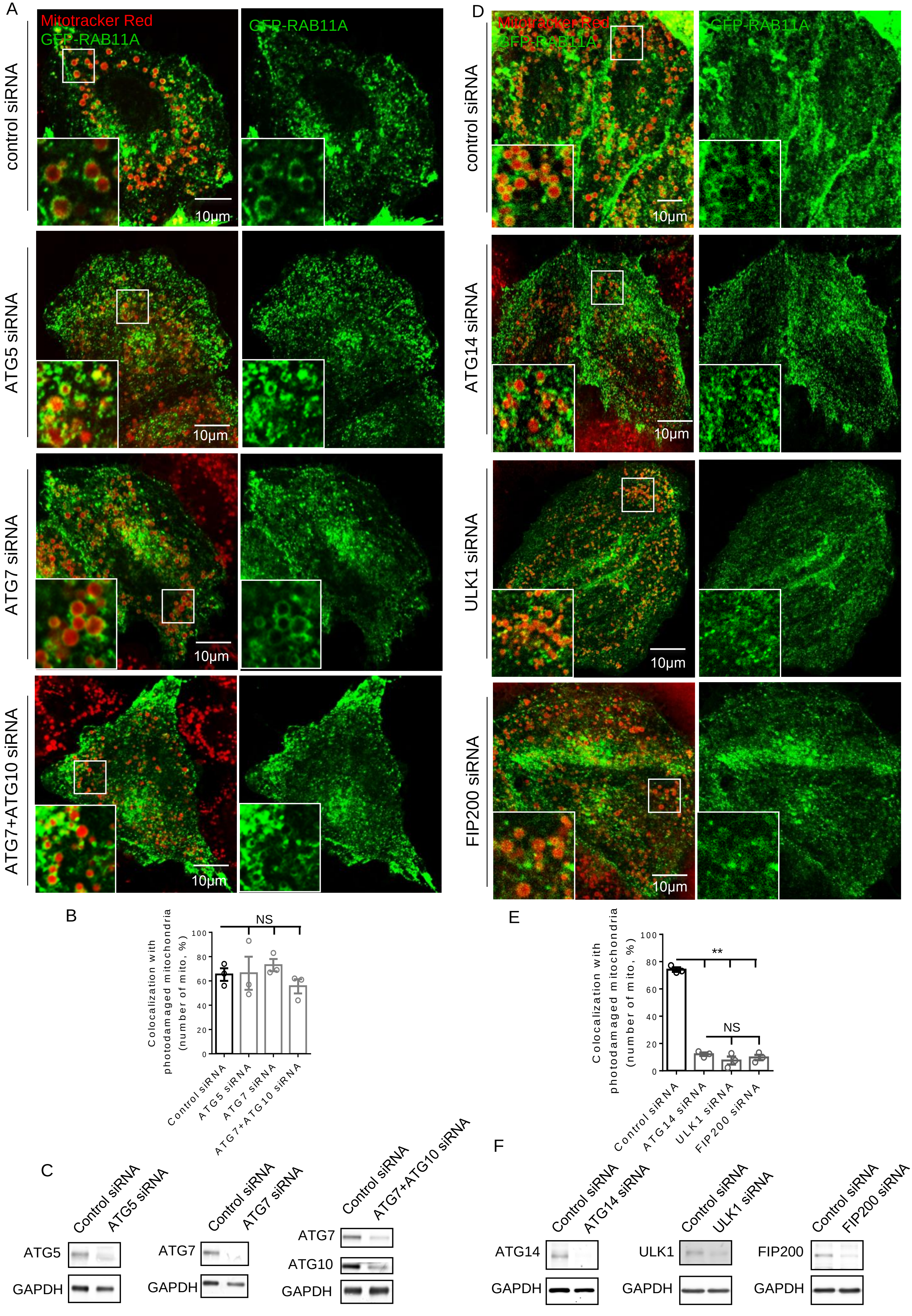

Puri, Vicinanza et al. Figure S7 (related to Figure 7): Damaged mitochondria are engulfed by RAB11A-positive compartment in cells silenced for late ATGs

Figure S7 (related to Figure 7): Damaged mitochondria are engulfed by RAB11A-positive compartment in cells silenced for late ATGs.A-C) HeLa cells were treated with control, or ATG5, or ATG7, or ATG7 and ATG10 siRNA for 5 days and transiently transfected with GFP-RAB11A (**A-B**) or processed for immunoblotting (**C**) to measure the degree of silencing. Cells were labeled with Mitotracker-Red and processed for live imaging (**A-B**). Mitochondria were photodamaged using 488-nm light. The percentage of photodamaged mitochondria engulfed by RAB11A in different conditions is shown in (**B**). Data are mean ± SEM (n=3 independent experiments, 10 cells counted for each condition in each experiments); One-way ANOVA with post hoc Tukey's test, NS=not significant.**D-F**) HeLa cells transfected with control or ATG14 or FIP200 or ULK1 siRNA for 5 days and transiently transfected with GFP-RAB11A (**D-E**) or processed for immunoblotting (**F**) to measure the degree of silencing. Cell treated and analysed as in (**A-B**). Data are mean ± SEM (n=3 independent experiments, 10 cells counted for each condition in each experiments); One-way ANOVA with post hoc Tukey's test, $** = p<0.01$, NS=not significant.
